# Heterogeneity of brain functional connectivity, transcriptome, and neurotransmitter profiles in major depressive disorder

**DOI:** 10.1017/S0033291725102171

**Published:** 2025-11-12

**Authors:** Qian Li, Haoran Li, Fenghua Long, Yufei Chen, Yitian Wang, Beisheng Yang, Melissa P. DelBello, Robert K. McNamara, Fei Li, Qiyong Gong

**Affiliations:** 1 Department of Radiology, Huaxi MR Research Center (HMRRC), Institution of Radiology and Medical Imaging, West China Hospital of Sichuan University, Chengdu, Sichuan Province, PR China; 2 Psychoradiology Key Laboratory of Sichuan Province, West China Hospital of Sichuan University, Chengdu, Sichuan Province, PR China; 3 Department of Psychiatry and Behavioral Neuroscience, University of Cincinnati College of Medicine, Cincinnati, OH, USA; 4 Xiamen Key Lab of Psychoradiology and Neuromodulation, Department of Radiology, West China Xiamen Hospital of Sichuan University, Xiamen, Fujian Province, PR China

**Keywords:** brain, cognition, gene, major depressive disorder, neurotransmitter, resting-state functional magnetic resonance imaging, subtype

## Abstract

**Background:**

Major depressive disorder (MDD) is a heterogeneous with underlying mechanisms that are insufficiently studied. We aimed to identify functional connectivity (FC)-based subtypes of MDD and investigate their biological mechanisms.

**Methods:**

Consensus clustering of FC patterns was applied to a population of 829 MDD patients from the REST-Meta-MDD database, with validity assessed across multiple dimensions, including atlas replication, cross-validated classification, and drug-naïve subgroup analysis. Regression models were used to quantify FC alterations in each MDD subgroup compared with 770 healthy controls, and to analyze spatial associations between FC alterations and publicly available gene transcriptomic and neurotransmitter receptor/transporter density databases.

**Results:**

Two stable MDD subtypes emerged: hypoconnectivity (*n* = 527) and hyperconnectivity (*n* = 299), which had both shared and distinct regions with remarkable FC alterations (i.e. epicenters) in the default mode network.

There were several common enriched genes (e.g. axon/brain development, synaptic transmission/organization, etc.) related to FC alterations in both subtypes. However, glial cell and neuronal differentiation genes were specifically enriched in the hypoconnectivity and hyperconnectivity subtypes, respectively.

Both subtypes showed spatial associations between FC alterations and serotonin receptor/transporter density. In the hypoconnectivity subtype, FC alterations correlated with GABA and acetylcholine receptor densities, while norepinephrine transporter and glutamate receptor densities were linked to the hyperconnectivity subtype.

**Conclusions:**

Our findings suggested the presence of two neuroimaging subtypes of MDD characterized by hypoconnectivity or hyperconnectivity, demonstrating robust reproducibility. The two subtypes had both shared and distinct genetic mechanisms and neurotransmitter receptor/transporter profiles, suggesting potential clinical implications for this heterogeneous disorder.

## Introduction

Major depressive disorder (MDD) is one of the most prevalent psychiatric disorders worldwide, with considerable heterogeneity in clinical symptoms and treatment outcomes (Cui et al., 2024). While this heterogeneity poses a substantial challenge for accurate diagnosis and prognosis, the underlying biological causes remain unknown. To address this, prior research has assigned MDD patients to subtypes based on clinical symptoms (Wu et al., 2022) or cognition (Hack et al., 2023). However, there is also growing interest in subtyping with neuroimaging biomarkers (Drysdale et al., 2017), especially functional connectivity (FC)-based subtyping. For example, Liang et al. (2020) identified two subtypes with distinct FC changes in the default mode network (DMN) areas, which was further supported by other works (Sun et al., 2023; Wang et al., 2021). However, these studies primarily analyzed data at the network level without exploring the transregional connectivity differences among MDD subtypes and healthy controls (HCs), and did not elucidate the critical brain regions underlying different subtypes.

Building on this gap, it is notable that the human brain operates through extensive interregional signaling, involving synchrony and coactivation. While this organization enables efficient communication, it also introduces vulnerability, as disruptions may co-occur with abnormalities among anatomically or functionally connected areas under pathological conditions (Zeighami et al., 2015). Among these regions, some may act as central nodes, where functional alterations are prominent both locally and in their connected areas, similar to a so-called epicenter, which may potentially play crucial roles in neurobiological pathogenesis in MDD. As a psychiatric disorder with widely reported dysconnectivity, different MDD subtypes may also show distinct patterns of FC alterations concentrated in such central regions. Although previous studies have identified MDD’s subtype-specific structural epicenters, such as cortical thinning or thickening in the frontal and parietal cortices (Li et al., 2024), few have explored functional epicenters based on FC, limiting our understanding of functional pathogenesis of MDD subtypes.

Another limitation of previous studies is that they classified patients with MDD without thoroughly investigating the underlying biological mechanisms. Given the multifactorial nature of MDD pathogenesis (Kamran, Bibi, Ur Rehman, & Morris, 2022), it is important to integrate these factors into assessments of MDD heterogeneity. The first factor is gene expression, which is significantly associated with both the pathogenesis and brain functional alterations in MDD (Liu, Abdellaoui, Verweij, & van Wingen, 2023). In view of the contribution of genetic factors to the pathoetiology of MDD, researchers have identified clinical subtypes with varied genetic correlations (from 0.55 to 0.86) within each subtype (Nguyen et al., 2022), suggesting genetic implications of MDD heterogeneity. However, the role of specific genetic factors on varied neuroimaging subtypes and FC alterations has not been fully elucidated. This can now be addressed using imaging transcriptomics (Arnatkeviciute, Markello, Fulcher, Misic, & Fornito, 2023) and the Allan Human Brain Atlas (AHBA; Hawrylycz et al., 2012), which contains comprehensive gene expression data and allows for the assessment of relationships between transcriptional profiles and neuroimaging data.

The dysregulation of various neurotransmitter systems has also been implicated in FC alterations and the excitation and inhibition (E/I) imbalance in MDD (Hu, Tan, Hirjak, & Northoff, 2023), supporting the use of antidepressants that targeting specific neurotransmitter systems (Mihaljević, Pavlović, Reiner, & Ćaćić, 2020). However, the treatment outcomes are inconsistent (Rost, Binder, & Brückl, 2023), indicating heterogeneous deficits in neurotransmitter systems among MDD patients. Using databases offering neurotransmitter system-density data (Markello et al., 2022), researchers can now investigate the associations between neurotransmitter and neuroimaging data. In addition, cognitive dysfunction is another critical characteristic of MDD, contributing to significant functional disability in MDD patients (Lam, Kennedy, McLntyre, & Khullar, 2014). Identifying cognitive domains associated with different subtypes by correlating comprehensive cognitive databases with neuroimaging data can therefore enhance the understanding of MDD subtypes. However, subtype-specific neurotransmitter bases and cognitive correlations have not been extensively explored.

**Figure 1.**
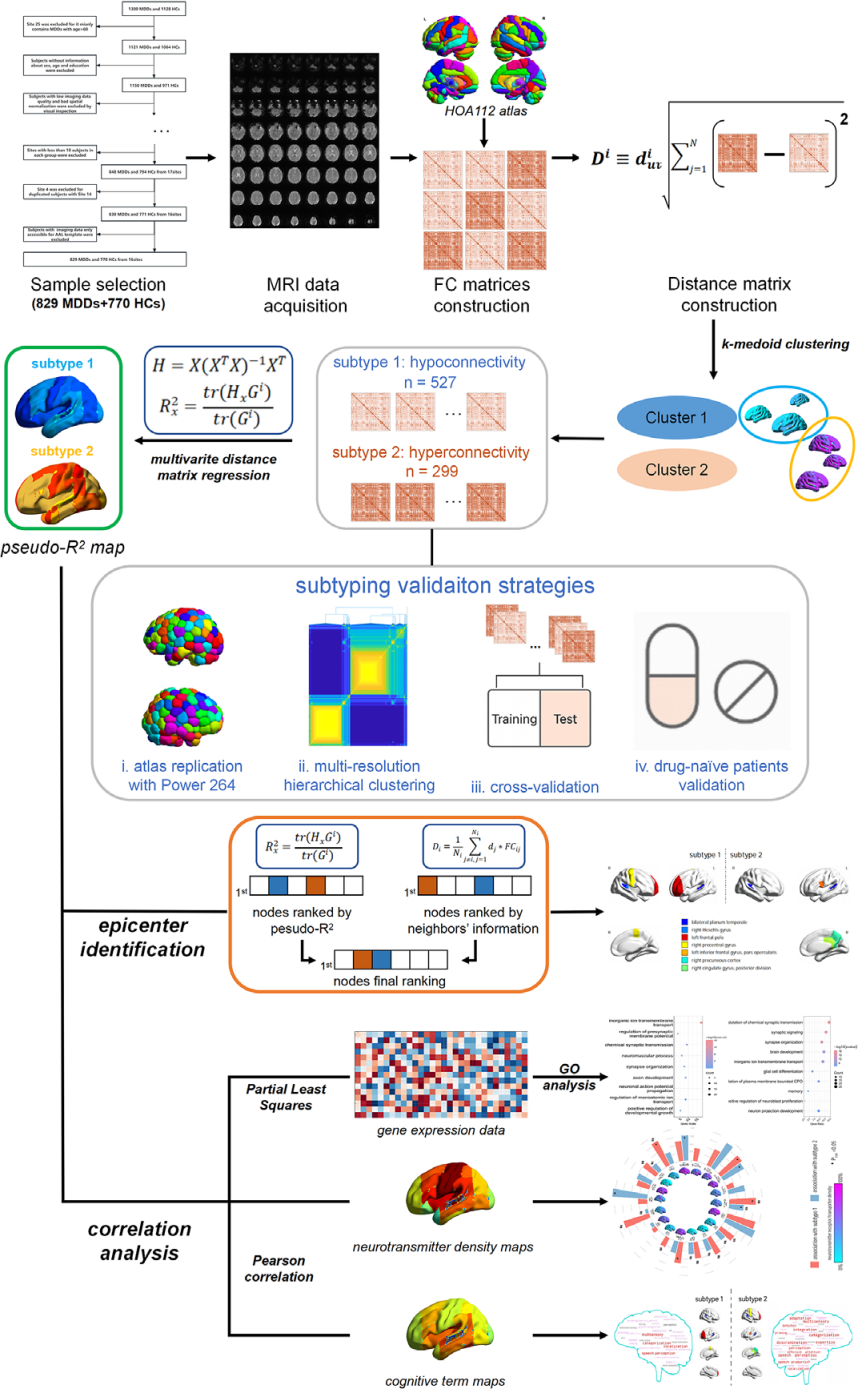
Study overview for identifying MDD subtypes and associated biological mechanisms. *Note:* GO, ‘gene ontology’; HCs, ‘healthy controls’; HOA, ‘Harvard-Oxford atlas’; FC, ‘functional connectivity’; MDD, ‘major depressive disorder’; MRI, ‘magnetic resonance imaging’.

Therefore, the primary objective of the present study was to: (1) identify specific MDD subtypes based on FC derived from resting-state functional magnetic resonance imaging (rs-fMRI) using a large multisite database (829 patients and 770 controls) with robust validations; (2) investigate the between-group differences in overall connectivity and identify the FC-based epicenter representing each subtype; and (3) explore the shared and specific associations between FC alterations and transcriptomics, neurotransmitter receptor/transporter, and cognition in each MDD subtype (Figure 1). We hypothesized that (1) FC-based MDD subtypes exist with variations in overall connectivity patterns compared with HCs; (2) the FC alterations in each subtype can be partly represented by epicenters belonging to different brain networks, such as DMN; and (3) some shared biological mechanisms related to the basic pathogenesis of MDD, such as abnormal intercellular connectivity, might be found in each MDD subtype, as well as different genetic, neurotransmitter, and cognitive correlations.

## Methods and materials

### Participants

A total of 1300 individuals diagnosed with MDD and 1128 HCs from 25 Chinese sites in the REST-meta-MDD consortium were initially considered. For each participant, demographic and clinical characteristics, including age, sex, education, illness duration, medication status, 17-item Hamilton Depression Rating Scale (HAMD), and Hamilton Anxiety Rating Scale (HAMA), were recorded. All participants provided written informed consent at their local institution. We followed the standard procedures provided by REST-meta-MDD consortium to conduct sample selection (Yan et al., 2019), with exclusion criteria including incomplete demographic information, age beyond 18–65 years, and poor image quality (Supplementary Figure S1). After selection, 829 MDD patients (523 female/306 male, mean age ± SD 34.4 ± 11.6 years) and 770 HCs (455 female/315 male, mean age ± SD 34.6 ± 13.2 years) from 16 sites were included (Supplementary Tables S1 and S2).

### MRI data preprocessing

High-resolution, three-dimensional, T1-weighted structural images and rs-fMRI of participants were obtained at their own local institution. The MRI scanning parameters for each site are provided in Supplementary Table S3. Imaging preprocessing was conducted by DPARSF software (rfmri.org/DPARSF), where time series from 112 Harvard-Oxford ROIs atlas (Makris et al., 1999) were extracted and FC between these ROIs was computed (see Supplementary Methods). ComBat was applied to harmonize our multisite imaging data before conducting subtyping to control scanner/site effects with empirical Bayes methods (Johnson, Li, & Rabinovic, 2007). The diagnostic label for each participant was specified as a biological variable of interest to protect the group-level FC differences during harmonization.

To evaluate site-related effects in the MRI data before harmonization, the Kruskal–Wallis test was used to determine whether the median FC values differed significantly across imaging sites (Lin et al., 2021), and Levene’s test was employed to assess variance homogeneity across sites (Soave & Sun, 2017). These tests allowed us to examine potential site-driven heterogeneity in FC patterns before data harmonization. These preharmonization tests revealed that 6190 FC exhibited significant median differences associated with site variation and 2771 FC showed significant variance heterogeneity; in contrast, following ComBat harmonization, the site effects have been effectively controlled, with post-harmonization analyses demonstrating non-significant inter-site differences across all whole-brain FC (*P* > 0.05), highlighting the efficacy of ComBat in controlling site-related biases.

### MDD subtyping and validation analysis

Consensus clustering was applied to characterize MDD subtypes. Age, sex, education, and head motion (i.e. framewise displacement [FD]) were regressed out from harmonized FC matrices (constructed using both positive and negative FC values) before clustering. Then, the distance between MDD patients *u* and *v* ( 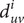 ) was calculated based on the Euclidean distances of FC between each ROI (*i*) and any other ROIs in the network:

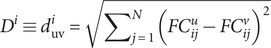

where *D^i^* denotes the distance matrix between each participant *i* 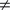 *j*, and *N* is the total number of ROIs (here is 112 in our study). Then, each distance matrix *D^i^* was partitioned into *k* clusters using *k*-medoids method, yielding a consensus matrix *C* for the current *k.* The *k* value corresponds to a different scale or resolution of clustering, that is, larger *k* results in more and smaller clusters, meaning higher resolution but less overall information. According to the optimizing strategy for capturing both large-scale and small-scale structural details, a final consensus matrix was evaluated by averaging all *C* values over the *k* range in the interval (2–20 in our study; Rasero et al., 2017). The final consensus matrix *C* was further partitioned into communities by Newman and Girvan-like modularity maximization (Newman & Girvan, 2004) to obtain an optimal output partition (i.e. FC-based MDD subtypes for the present study) that maximized the network modularity. Bootstrapping was applied to assess the stability of each subtype and the 95% confidence intervals (CIs) of the estimated maximum modularity which reflects the statistical difference from zero.

To evaluate the robustness of our subtyping solution, we further validated our results across multiple strategies (Figure 1), including (1) replication using a high-resolution brain atlas (i.e. Power-264); (2) alternative clustering method via multiresolution hierarchical clustering, given the resolution dependence of clustering algorithms (Reichardt & Bornholdt, 2006); (3) cross-validated support vector machine prediction of subtype labels with matched halves generated by stratified sampling on demographic and head motion variables; and (4) replication in a first-episode, drug-naïve MDD cohort to exclude medication effects (Yan et al., 2019). The details of MDD subtyping and validations are provided in Supplementary Methods.

### Clinical and FC characteristics of MDD subtypes

After we found robust FC-based MDD subtypes, the differences in age, education, illness duration, HAMD, and HAMA total scores between MDD subtypes (two subtypes identified in the present study; see Results) were tested by two-sample *t*-test, while sex, episode status, and medication by chi-square test. Multiple linear regression was applied to assess the differences in the overall connectivity per participant between (1) the whole MDD group and HCs and (2) each MDD subtype and HCs group with age, sex, education, and FD value as covariates. The overall connectivity for each participant was defined as the average value of both positive and negative FC of the abovementioned harmonized connectivity matrix in the main analysis. To ensure robustness of this comparison, we additionally computed overall connectivity using alternative metrics, including the median of all FC values, the 10% trimmed mean (i.e. excluding the top and bottom 10% of values), the mean of all positive FC values, and the mean of absolute FC values. A significance *P*-value of 0.05 was set for all the clinical and overall connectivity comparisons.

### Epicenter identification

After subtyping, multivariate distance matrix regression (MDMR; Shehzad et al., 2014) was first applied to quantify the FC alterations in all brain regions. MDMR was used for calculating a pseudo-*R* ^2^ effect size, where a higher pseudo-*R* ^2^ suggested greater FC alterations (see Supplementary Methods). After calculating pseudo-*R* ^2^ in every ROI, we attained pseudo-*R* ^2^ maps for each subtype group, which will be used for subsequent identification of epicenters and correlation analyses.

Following the epicenter identification approach as a prior work did (Shafiei et al., 2020), we defined a brain region as a potential epicenter if it showed large FC alterations (pseudo-*R* ^2^) and its FC-connected neighbors also exhibited high alterations. Neighbor influence was quantified as the weighted mean FC alteration of connected regions (see Supplementary Methods). Regions were ranked separately by their own pseudo-*R* ^2^ and by neighbors’ alterations, and those ranked highly on both lists were tested for significance via spatial permutation (spin test; 1000 iterations, *P* < 0.05). All significant epicenters were reported without additional top-*N* criteria.

### Biological characteristics of MDD subtypes

#### Transcriptomics

Brain gene transcription data were obtained from AHBA (Hawrylycz et al., 2012). The dataset was derived from six healthy postmortem adult brains, including more than 20,000 gene expression data at 3702 brain regions. We mapped the gene expression data to the 112 ROIs of Harvard-Oxford template using abagen toolbox (Markello et al., 2021), with a proposed preprocessing pipeline (see Supplementary Methods). After preprocessing, there were 15,633 genes survived. As AHBA includes genes expressed in any body tissue, we focused on the 1920 brain-specific genes, relative to other tissues, extracted from the Human Protein Atlas (www.proteinatlas.org). The precise list of these 1920 brain-specific genes is provided in Supplementary Table S5.

#### Associations between FC alterations and transcriptomics

Because only two of the six AHBA donors had bilateral sampling, we restricted our association analysis between gene expressions and FC alterations (i.e. pseudo-*R* ^2^ map) in the 56 ROIs to the left hemisphere data. Partial least squares (PLS) regression was used to estimate the association between FC alterations in each MDD subtype relative to HCs and the gene expression measurements for 1920 brain-specific genes. Among all components, the first (PLS1) showed the strongest correlation with the FC alteration map and explained the largest variance (approximately 28.5%). To mitigate false positives from spatial autocorrelation in the gene expression data, we followed the recommendations of (Fulcher, Arnatkeviciute, & Fornito, 2021), in which the statistical significance of PLS1 was tested by permuting the response variables (FC alterations map, here) 1,000 times to build a null distribution while keeping the genetic expression matrix unchanged. The *P* value was estimated as the percentage of null correlations that exceeded the primary correlations between PLS1 and FC alterations map estimated on the original data. The genes with PLS1 weights *P*_FDR_ < 0.05 were set as significant and were divided into two groups (PLS1+ and PLS1−) according to their positive or negative correlation coefficients, which were used for subsequent analyses (details in Supplementary Methods).

To gain deeper insights into the functional roles of these genes, gene ontology (GO) analyses and hub genes identification were performed separately for significant genes in PLS1+ and PLS1− in each subtype. GO biological process enrichment analysis was conducted using Metascape toolbox (Zhou et al., 2019) with *P*_FDR_ < 0.05 set as significant, and the top 10 significant GO terms were reported here. Next, to identify the hub genes, we first constructed the protein–protein interaction networks with the significant genes and then calculated maximal clique centrality (MCC) values reflecting network centrality for each gene (see Supplementary Methods). Subsequently, the top three MCC-ranked genes in each network were selected as hub genes and reported.

#### Associations between FC alterations and neurotransmitter receptor/transporter

Neurotransmitter receptor/transporter density maps were downloaded with neuromap toolbox (Markello et al., 2022). The maps with the same neurotransmitter receptor/transporter were averaged after scaling between 0 and 1, and then we mapped those brain maps to the Harvard-Oxford atlas for subsequent association analysis. Eighteen items of neurotransmitter receptor/transporter were finally extracted (Supplementary Table S7). Pearson correlation analysis was used to explore the association between FC alterations of each MDD subtype and neurotransmitter receptor/transporter. Instead of PLS, Pearson correlation was used for neurotransmitter and subsequent cognitive associations because the number of neurotransmitter density maps and cognitive terms was relatively small (*n* = 18 and 124, respectively), and each variable was biologically interpretable, allowing for a direct spatial correspondence analysis with FC alterations. Spin permutation tests (1,000 times) were applied to examine the statistical significance of correlations (Alexander-Bloch et al., 2018). Statistically significant correlations were set as *P*_FDR_ < 0.05.

#### Associations between FC alterations and cognition

We also explored the association between FC alterations of each MDD subtype and 124 cognitive domains’ maps derived from Neurosynth and Cognitive atlas (http://neurosynth.org/; http://cognitiveatlas.org/) databases using Pearson correlation with spin permutations test (see Supplementary Methods).

## Results

### MDD subtypes

The modularity of consensus clustering, derived from bootstrapping significantly differed from zero (0.168, 95% CI 0.158–0.178), indicating the existence of MDD subtypes, where two stable MDD subtypes were further identified: 527 patients (63.57% of all patients with MDD) were assigned to subtype 1, and 299 patients (36.07%) were assigned to subtype 2. The two major subtypes were stably replicable during bootstrapping with the stability score of 0.946 and 0.821 for each subtype, respectively. In addition to these two main subtypes, there were three residual subtypes (0.36%), each with only one participant and stability scores all <0.5 (0.049, 0.137, and 0.235, respectively), suggesting low consistency and replicability, which were excluded for further analyses.

We validated our subtyping results in varied aspects and found high stability scores in all subtypes in validation analyses (from 0.75 to 0.989), high similarities between validation and our primary results (from 0.6 to 0.77), and a high predictive accuracy (95.7%) in a machine-learning validation analysis (details in Supplementary Results and Supplementary Figure S2).

### Clinical and FC characteristics, and epicenters of each subtype

There were no significant differences in age, sex, education, illness duration, HAMD and HAMA total scores, or episode status between the two MDD subtypes, but subtype 2 included a higher proportion of drug-naïve participants than subtype 1 (Table 1).

**Table 1.** Demographic and clinical characteristics of two subtypes of major depressive disorder in our study

Variable	Subtype 1	Subtype 2	*P* value^d^
Sample size, *N*	527	299	–
Age, years, mean ± SD	34.1 ± 11.6	34.9 ± 11.5	0.373
Sex, *N*, female/male	331/196	190/109	0.833
Education, years, mean ± SD	12.0 ± 3.3	11.8 ± 3.4	0.365
Head motion (FD), mm, mean ± SD	0.066 ± 0.037	0.071 ± 0.034	**0.036**
Illness duration^a^, months, mean ± SD	38.7 ± 59.4	39.8 ± 64.5	0.828
HAMD total score^b^, mean ± SD	20.9 ± 6.6	21.9 ± 6.7	0.061
HAMA total score^c^, mean ± SD	19.1 ± 9.4	19.6 ± 8.6	0.576
Episode status			
First-episode, *N* (%)	259(49.1%)	156(52.2%)	0.279
Recurrent, *N* (%)	139(26.4%)	69(23.1%)	
Unknown, *N* (%)	129(24.5%)	74(24.7%)	
Medication status			
Drug-naive, *N* (%)	183(34.7%)	130(43.5%)	**0.005**
On medication, *N* (%)	154(29.2%)	65(21.7%)	
Unknown, *N* (%)	190(36.1%)	104(34.8%)	

Abbreviations: FD, framewise displacement; HAMD, Hamilton Depression scale; HAMA, Hamilton Anxiety scale.

a Data were available for 452 participants for subtype 1, and 242 participants for subtype 2.

b Data were available for 467 participants for subtype 1, and 275 participants for subtype 2.

c Data were available for 326 participants for subtype 1, and 193 participants for subtype 2.

d *P* value was calculated by two-sample *t* test or chi-square test. Bold values indicate statistically significant differences (p < 0.05).

**Figure 2.**
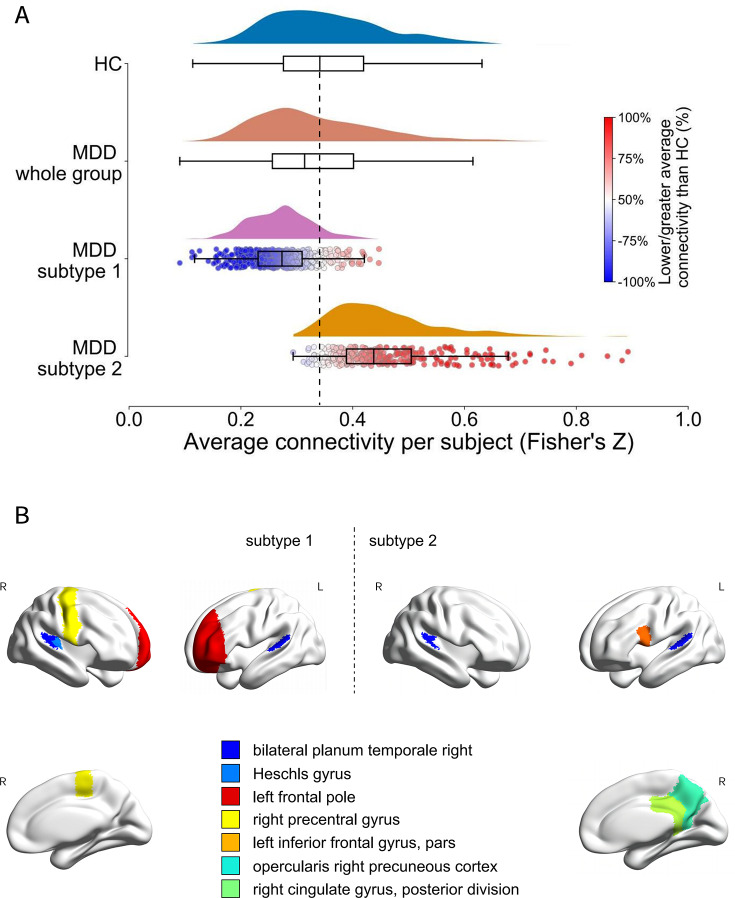
Two stable neurophysiology major depressive disorder (MDD) subtypes and epicenters of each subtype were found. Subtype 1 was characterized by hypoconnectivity and subtype 2 by hyperconnectivity. (A) Box and histogram plots of the individual average connectivity values (measured as Fisher’s *Z*) for the healthy controls (HCs) group (blue), all participants with MDD without subtyping (brown), and two MDD subtypes (pink and orange). The median value of the HCs group was marked as the connectivity baseline by a dashed vertical line. Values above the baseline corresponded to hyperconnectivity and those below the baseline corresponded to hypoconnectivity. For subtypes 1 and 2, cool and warm colors were used for participants to show within-group hypoconnectivity and hyperconnectivity relative to HCs, respectively. (B) Brain epicenter maps of two MDD subtypes. *Note:* L, ‘left’; R, ‘right’.

Compared with HCs, the whole MDD group (*β* = −0.0125, *t*_1593_ = −2.689, *P* = 0.007) and subtype 1 patients (*β* = −0.085, *t*_1291_ = −17.014, *P* < 0.001) showed significant overall hypoconnectivity, whereas subtype 2 patients showed significant hyperconnectivity (*β* = 0.100, *t*_1063_ = 14.106, *P* < 0.001; Figure 2A). When different FC metrics defining overall connectivity were used, the same connectivity patterns of the whole group and each subtype were observed (Supplementary Table S4).

Nominally significant correlations (*P* < 0.05) were found between FC and symptom scores in both MDD subtypes. For HAMD, specifically, subtype 1 had 232 associated FCs and subtype 2 had 298, with 13 overlaps. For HAMA, subtype 1 had 387 FCs and subtype 2 had 163, with only eight overlaps. Pearson correlation analysis revealed shared cognitive classifications associated with FC alterations for both subtypes in language, reasoning and decision-making, perception, and multisensory, but subtype 2 was specifically associated with learning and memory, and attention (details in Supplementary Results, Supplementary Tables S8 and S9, and Supplementary Figure S3).

Overlapping epicenters of the two identified subtypes were found in the bilateral planum temporale (PT). Additionally, distinct epicenters of subtype 1 were found in the left frontal pole, right precentral gyrus, and Heschl’s gyrus, whereas distinct epicenters of subtype 2 were found in the left pars opercularis of the inferior frontal gyrus, right posterior cingulate gyrus, and precuneus (Figure 2B).

### Associations between FC alterations and transcriptomics

**Figure 3.**
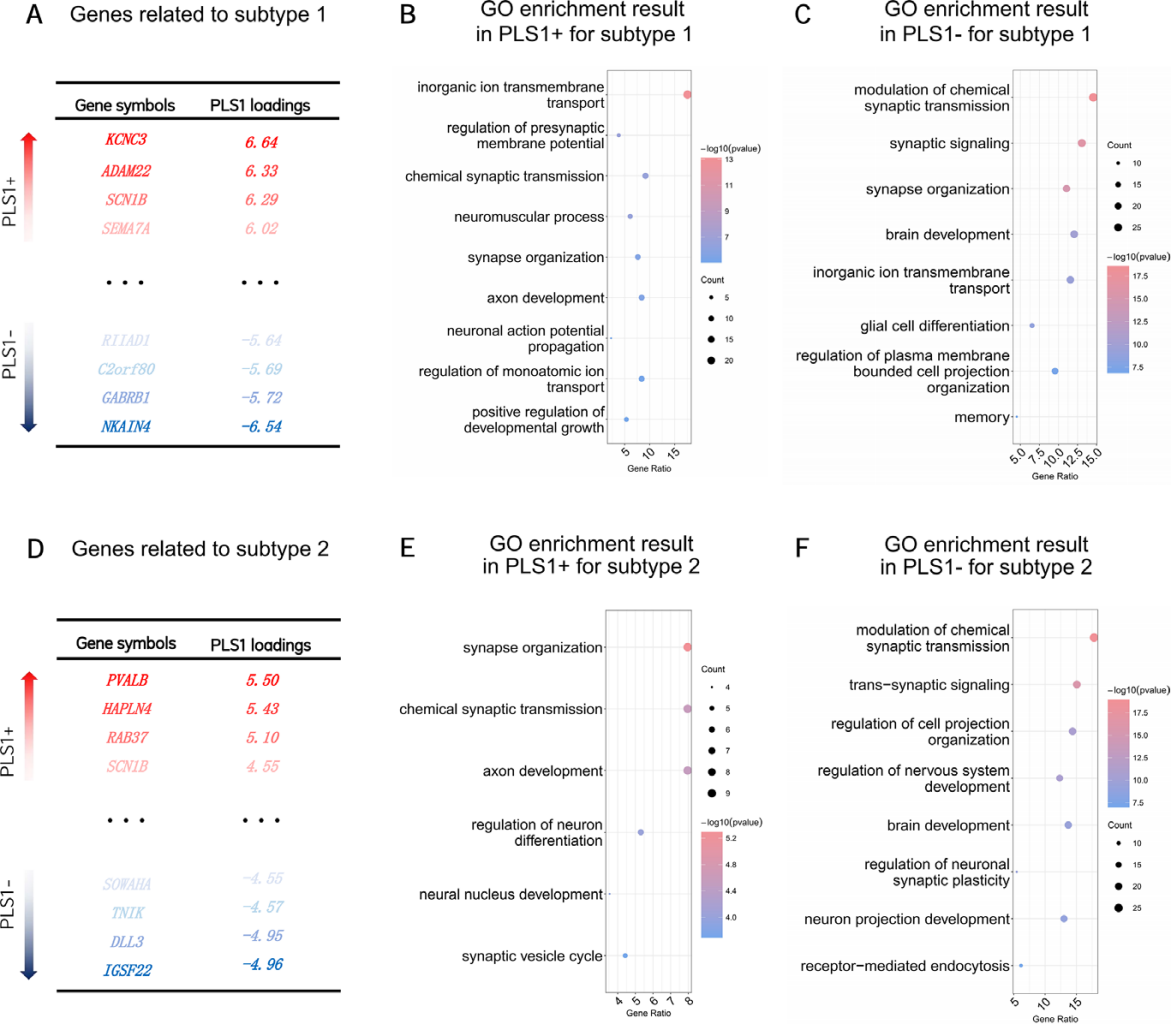
Associations between functional connectivity alterations and transcriptomics for the two subtypes of major depressive disorder. Panels A and D listed the PLS1+ and PLS1− genes for subtypes 1 and 2, respectively. Panels B, C, E, and F were the bubble plots of the GO enrichment results for PLS1+/PLS1− genes for each subtype. The vertical coordinate represented the corresponding GO set, while the horizontal coordinate represented the ratio of significant genes found by PLS to the total number of genes in this GO set. The size of the bubble represented the significant gene counts of the corresponding PLS set, while color of which was related to the −log_10_ (P). *Note:* AC, ‘adenylate cyclase’; CPO, ‘cell projection organization’; GO, ‘gene ontology’; GPCR, ‘G protein-coupled receptor’; PLS, ‘partial least squares’.

The FC alteration patterns in both MDD subtypes were associated with brain gene expression profiles (subtype 1, *r* = 0.40, *P*_perm_ = 0.002; subtype 2, *r* = 0.49, *P*_perm_ = 0.001). For subtype 1, 132 genes with positive weights and 199 with negative weights in the PLS1 reached statistical significance (*P*_FDR_ < 0.05, Figure 3A), while for subtype 2, 113 genes with positive weights and 146 with negative weights in PLS1 reached statistical significance (*P*_FDR_ < 0.05, Figure 3D).

The shared GO enrichment functions of significant genes in both subtypes included transmembrane transport, synaptic transmission/organization, and regulation of neurotransmitters, axon/brain development, and cell projection. Glial cell differentiation was specifically enriched in subtype 1, whereas neuron differentiation was specific to subtype 2 (details in Figure 3 and Supplementary Results). The shared hub genes’ function in both subtypes was related to membrane potential and neurotransmitter release. Genes related to GABA receptors were specifically found in subtype 1, while genes related to glutamate receptors were found in subtype 2 (details in Supplementary Results and Supplementary Table S6).

### Associations between FC alterations and neurotransmitter receptor/transporter

**Figure 4.**
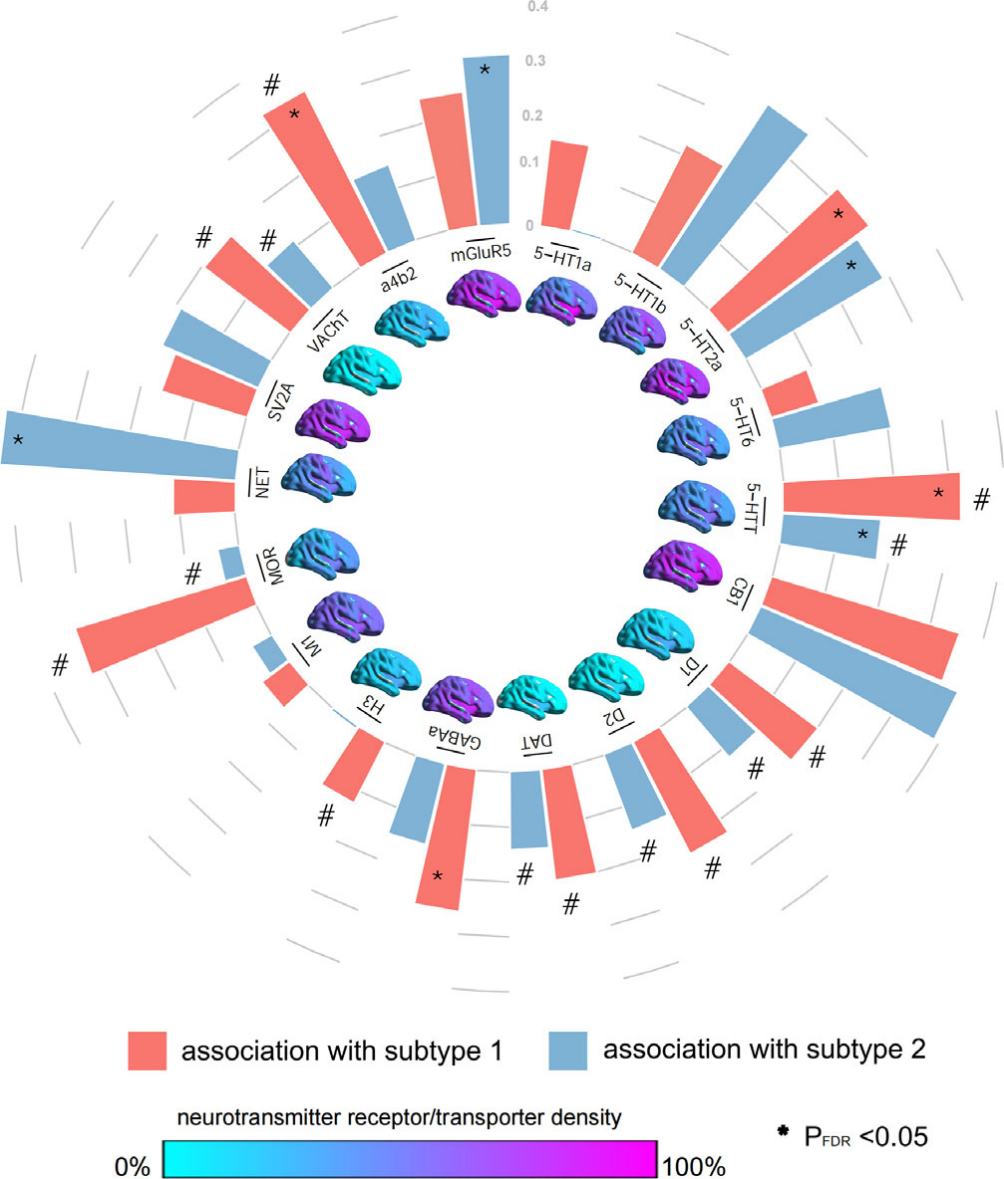
Associations between functional connectivity alterations and neurotransmitter receptor/transporter for two subtypes of major depressive disorder. In the center of the circle, there were the density maps of each neurotransmitter receptor/transporter. The circular bar plot showed the correlations between neurotransmitter receptor/transporter and subtype 1 (red) and subtype 2 (blue), respectively. The height of the bars represented the correlation coefficients (*r*), and the *r* values are listed in Supplementary Table S7. We marked * or # on the top of the bars to show if the correlation survived the false-discovery rate (FDR) correction or *r* was negative (i.e. *r* < 0).

Densities of serotonin receptor (5-HT2a: subtype 1, *r* = 0.32, *P*_FDR_ = 0.009; subtype 2, *r* = 0.288, *P*_FDR_ = 0.018) and serotonin transporter (5-HTT: subtype 1, *r* = −0.321, *P*_FDR_ = 0.009; subtype 2, *r* = −0.176, *P*_FDR_ = 0.03) spatially correlated with FC alterations in both MDD subtypes. Densities of GABA receptor (GABAa, *r* = 0.258, *P*_FDR_ = 0.012) and acetylcholine receptor (a4b2, *r* = −0.328, *P*_FDR_ = 0.032) were spatially correlated with FC alternations only in subtype 1, while norepinephrine transporter (NET, *r* = 0.427, *P*_FDR_ = 0.018) and glutamate receptor (mGluR5, *r* = 0.306, *P*_FDR_ = 0.041) were correlated with FC alternations only in subtype 2 (Figure 4 and Supplementary Table S7).

## Discussion

Our findings revealed two FC-based MDD subtypes. Compared with HCs, subtype 1 was characterized by hypoconnectivity, whereas subtype 2 was characterized by hyperconnectivity. These subtypes had shared epicenters and distinct ones, some of which were located in the DMN. FC alterations in both subtypes correlated with intercellular communication and membrane potential, serotonin receptor/transporter, and multiple cognitive domains. However, FC alterations for subtype 1 exhibited spatial associations with glial cell differentiation and GABA and acetylcholine receptors, whereas FC alterations for subtype 2 specifically correlated with neuronal differentiation, norepinephrine transporters and glutamate receptors, as well as cognition of learning and memory, and attention.

### Overall FC patterns

We found that, while the whole MDD group exhibited hypoconnectivity compared to controls, this pattern was driven by only one subtype. The opposing hyperconnectivity pattern in a second subtype was masked by the group-level analysis. This demonstrates that averaging across all MDD patients obscures fundamental FC heterogeneity and underscores the necessity of subtyping to reveal neurobiologically distinct subgroups. Furthermore, the two MDD subtypes with opposite overall FC patterns did not exhibit dramatically different clinical profiles, which suggested that this neurobiological difference may not be fully captured by traditional clinical assessments, possibly due to divergent biological mechanisms (Wang et al., 2021). Despite the absence of clinical differentiation, the neurobiological differences revealed by FC patterns could guide the development of more nuanced diagnostic criteria that better capture the underlying heterogeneity of MDD; this idea was corroborated by the varied cognitive correlations observed for each subtype in our findings.

The distinct FC patterns observed in the two MDD subtypes may reflect divergent underlying neurobiological processes from a neurodevelopmental perspective. For example, hypoconnectivity in subtype 1 could indicate delayed or impaired neuroplasticity with reduced synaptic efficiency or axonal connectivity (Price & Duman, 2020). Indeed, the GO enrichment analysis in our study also revealed associations for subtype 1 with biological processes such as axon development and brain development, indicating that these processes could be crucial to the pathogenesis for this subtype. In contrast, hyperconnectivity in subtype 2 may result from overcompensation for neurodevelopmental disruptions, with pathological strengthening of connections between certain regions to offset deficits (Wade-Bohleber et al., 2020). This could lead to heightened emotional reactivity, a symptom often observed in MDD (Burkhouse et al., 2017), linking altered FC and depression symptoms in this subtype.

### FC epicenters

The identified functional alteration epicenters for each MDD subtype provided more specific FC indicators. In particular, PT, the shared epicenter for both subtypes, has been implicated in auditory function and related cognitive processes, such as speech perception (J. Jiang et al., 2024; Ramos Nuñez, Yue, Pasalar, & Martin, 2020), a cognitive domain found to be significantly associated with both subtypes in our study. These findings suggest that the FC alterations in PT may contribute to auditory symptoms observed in some MDD patients, such as auditory verbal hallucinations (Toh, Thomas, & Rossell, 2015). In addition, the cognitive control circuit summarized by Williams (Williams, 2016), which covers epicenters of both subtypes (i.e. the precentral gyrus for subtype 1 and the inferior frontal cortex for subtype 2), could be a key neural circuit for both subtypes. This idea is consistent with our findings showing the two subtypes’ significant association with cognitive functions, highlighting the crucial roles of these regions in the development of cognitive deficits in MDD patients.

Subtype-specific epicenters were also identified. For subtype 1, epicenters located in the frontal pole and Heschl’s gyrus (a subregion of superior temporal gyrus [STG]), and previous studies have also reported decreased FC in the STG and prefrontal regions in depression patients at early stage (Zou et al., 2016), suggesting that these regions may be critically involved in not only central but also early functional changes in MDD pathogenesis. However, findings related to STG have not been consistent across the literature, with both increased and decreased FC reported (Pan et al., 2020). This inconsistency could be attributed to the small sample size, clinical heterogeneities, and so forth; however, our identification of PT (a STG’s subregion) as a shared epicenter may help reconcile these discrepancies, as FC alterations may commonly be concentrated in PT across patients, but the direction of alteration varies between subtypes, contributing to heterogeneity in network-level findings.

In subtype 2, the identified epicenters were located primarily in the inferior frontal cortex and DMN regions, including the posterior cingulate cortex and precuneus. Prior studies have consistently reported increased FC in the inferior frontal areas among MDD patients (Xiao et al., 2024; Zhang et al., 2022), and both heightened FC and cortical thickening in DMN regions have also been observed (van Eijndhoven et al., 2013; Zhang et al., 2022). These findings may suggest compensatory proliferation along with increased FC in DMN regions in MDD patients, supporting the abovementioned idea of overcompensation for mental regulation in this subtype, as the DMN is vital in neurophysiological processes such as cognition, emotion, memory, and attention (Berman et al., 2011). These crucial roles of the DMN may also contribute to the specific cognitive correlations found for this subtype, as they can be classified into learning and memory, and attention, highlighting the importance of epicenters in understanding MDD pathogenesis.

### Genetic mechanisms

Our enrichment analysis revealed that the biological processes shared by these two subtypes were related to the development and function of axons and of the brain, cell projections, and chemical/electrical signal conduction. Among these processes, the importance of electrical signal conduction was further corroborated by the shared hub genes encoding potassium (KCNA1 and KCNAB3) and sodium (SCN1A and SCN1B) channels. These findings were in line with prior studies highlighting the roles of cellular dysconnection, brain development, and electrophysiological properties in the pathogenesis of MDD (Kamran et al., 2022).

Despite these shared genetic mechanisms, these two subtypes showed notable differences. In subtype 1, the GO enrichment results indicated that FC alterations might be influenced by genes associated with glial cell differentiation. One possible explanation is that dysregulation of glial differentiation, which contributes to neuronal pathology in MDD (Öngür, Bechtholt, Carlezon, & Cohen, 2014), damages neural activity, potentially leading to connectivity impairments. Another possible explanation involves the dysfunction of glial cell communication via gap junctions, which are crucial for regulating electrical synapses and brain development (H. Jiang, Zhang, Wang, & Chen, 2023), indicating that disruptions in these processes may further contribute to the pathogenesis of subtype 1. In contrast, the gene enrichment results identified the neuronal differentiation process as associated with subtype 2. The abnormal activation of such regulation may cause excessive neuronal regeneration (An et al., 2022), potentially contributing to increased FC in this subtype. Moreover, the dysregulation of neuronal differentiation may also be associated with impaired natural mechanisms for synaptic pruning, where excess connections are typically eliminated during development (Goda & Davis, 2003), contributing to hyperconnectivity.

### Neurotransmitter mechanisms

We found that serotonin receptors/transporters were spatially associated with FC alterations in both MDD subtypes, which reinforced the long-standing monoamine hypothesis of depression (Hirschfeld, 2000). Despite the distinct FC patterns observed in each subtype, the common involvement of the serotonergic system suggested that serotonin dysregulation may serve as a common pathway across the various presentations of MDD. This finding could have significant therapeutic implications, as traditional treatments targeting the serotonergic system, such as selective serotonin reuptake inhibitors (Mace & Taylor, 2000), may still be effective in most patients with MDD.

However, subtype-specific neurotransmitter systems beyond serotonin suggested by our findings may provide insight into the inconsistent treatment outcomes often observed in MDD patients (Rost et al., 2023) and inform targeted treatment strategies. For example, serotonin-norepinephrine reuptake inhibitors or ketamine could be particularly effective for patients in subtype 2, of which FC alterations were uniquely associated with norepinephrine transporter and glutamate receptor. Besides, recent studies have proposed a 5-HT-glutamate/GABA long circuit for rapid regulation of E/I imbalance (Li, 2020). Interestingly, given further support by the identified hub genes (e.g. GABBR for subtype 1, whereas GRIA1 for subtype 2), the dysregulation of the GABA and glutamate systems may be specific to subtypes 1 and 2, respectively, further indicating more targeted and precise efficacy in patients if these findings were used for treatment references for each subtype.

Our findings of distinct neurotransmitter-subtype correlations, together with prior work on neurotransmitter modulation of FC, suggest potential neurochemical bases for the observed alterations. In some networks, GABAergic activity tends to correlate negatively with FC (Chen et al., 2019), whereas noradrenergic and glutamatergic systems show positive correlations (Kapogiannis, Reiter, Willette, & Mattson, 2013; Ruggiero et al., 2021). Based on these findings, the hypoconnectivity observed in subtype 1 could be linked to the upregulation of functions in the GABAergic system, whereas hyperconnectivity in subtype 2 may correspond to the upregulation of norepinephrinergic and glutamatergic systems. However, acetylcholinergic–FC associations can be bidirectional (Keeley et al., 2022; Ruggiero et al., 2021), and the relationship between the glutamatergic system (e.g. mGluR5) and FC is inconsistent; they are positively correlated within MDD patients but negatively correlated within healthy individuals (Kim et al., 2019). These complexities further underscore the multifaceted nature of the relationship of neurotransmitter systems with FC alterations, providing more insights into MDD heterogeneity.

### Limitations

This study has limitations that should be considered. Methodologically, the inability to control for covariates such as MDD episodes, medication status, or psychosis comorbidity due to incomplete data (e.g. only 10 out of 16 sites included in our study offered comprehensive information on medication use for all participants) may have introduced bias. Similarly, the lack of cognitive-scale data directly from the participants limits the accuracy of the cognitive associations explored. Although we performed internal validation using cross-validation strategies, external validation with independent datasets is necessary to assess the generalizability of the identified subtypes. Regarding imaging transcriptomics, gene expression data were derived from a small number of healthy donors (*n* = 6) of different ethnic backgrounds (Hispanic and Caucasian) from our Asian sample, potentially introducing demographic bias. Additionally, only left-hemisphere data were used. Importantly, AHBA reflects normative expression and lacks patient-specific profiles, limiting our ability to capture individual variability. Future studies incorporating subject-level genetic or transcriptomic data and multiple MRI modalities (Li et al., 2023; Wang et al., 2024, 2025; You et al., 2022) will be valuable for directly and comprehensively exploring and validating the molecular distinctions between MDD subtypes.

## Conclusions

We identified two FC-based MDD subtypes exhibiting divergent overall FC patterns with both unique and shared epicenters in critical brain networks such as the DMN. By exploring genetic, neurotransmitter, and cognitive associations, we have further provided insights into the complex underlying mechanisms of MDD heterogeneity and highlighted the importance of considering FC-based subtypes in understanding the neurobiological heterogeneities of MDD. More broadly, such findings may inform the development of more targeted and precise treatments for patients in each MDD subtype.

## Supporting information

Li et al. supplementary material Li et al. supplementary material

## Data Availability

The data that support the findings of this study are available from the corresponding authors upon reasonable request.

## Supplementary material

The supplementary material for this article can be found at http://doi.org/10.1017/S0033291725102171.
