# Supplementary material for: Heterogeneity of brain functional connectivity, transcriptome, and neurotransmitter profiles in major depressive disorder
Source: Psychol Med. 2025 Nov 12;55:e341. doi: 10.1017/S0033291725102171 (PMC13058663; doi:10.1017/S0033291725102171)
Supplement: Li et al. supplementary material [file S0033291725102171sup001.docx]

**Heterogeneity in brain functional connectivity, transcriptome, and neurotransmitter profiles in major depressive disorder**

Supplementary Materials

[Supplementary methods 3](#_Toc22116)

[MRI data preprocessing 3](#_Toc27592)

[MDD subtyping and validation analysis 3](#_Toc26310)

[Clinical and FC characteristics of MDD subtypes 5](#_Toc7306)

[Epicenter identification 6](#_Toc32730)

[Biological characteristics of MDD subtypes 7](#_Toc3655)

*[Transcriptomics](#_Toc10074)* [7](#_Toc10074)

*[Associations between FC alterations and transcriptomics](#_Toc1730)* [8](#_Toc1730)

*[Associations between FC alterations and cognition](#_Toc16787)* [10](#_Toc16787)

[Supplementary results 10](#_Toc15150)

[Validation for MDD subtypes 10](#_Toc28839)

[Associations between FC alterations and transcriptomics 11](#_Toc18451)

[Associations between FC alterations and cognition 12](#_Toc32309)

[Table S1. Demographic characteristics for participants in each institution included in our study. 14](#_Toc31434)

[Table S2. Demographic and clinical characteristics of all participants included in our study. 15](#_Toc27534)

[Table S3. MRI scanning parameters for each site 16](#_Toc23404)

[Table S4. The differences in overall functional connectivity between the whole each patient subtype and controls, respectively, which was calculated by different definitions to validate the main results. 17](#_Toc9280)

[Table S5. The list of brain-specific genes. 18](#_Toc9127)

[Table S6. The hub genes, their maximal clique centrality (MCC) values, and their annotations for each subtype. 26](#_Toc28218)

[Table S7. Associations between spatial patterns of aberrant functional connectivity in each subtype of major depressive disorder and neurotransmitter receptor/transporter densities. 27](#_Toc28891)

[Table S8. Associations between spatial patterns of aberrant functional connectivity patterns and meta-analytic cognitive functions. 28](#_Toc19419)

[Table S9. Shared and subtype-2-specific cognitive classifications, and corresponding terms&annotations belonging to them. 31](#_Toc13410)

[Figure S1. Sample selection. 32](#_Toc26534)

[Figure S2. The two major MDD subtypes were identified by multi-resolution hierarchical clustering. 33](#_Toc15103)

[Figure S3. Associations between functional connectivity (FC) alterations and cognitions for two subtypes of major depressive disorder. . 34](#_Toc7797)

[References: 35](#_Toc11452)

**Supplementary methods**

**MRI data** **preprocessing**

High-resolution three-dimensional T1-weighted structural images and rs-fMRI of participants were obtained at their own local institution. The MRI scanning parameters for each site are provided in Table S3. Imaging preprocessing was conducted by using DPARSF software ([rfmri.org/DPARSF](https://rfmri.org/DPARSF)), primarily including slice-time correction, realignment, normalization, and nuisance (including nuisance signals and head motion during scanning quantified by framewise displacement [FD]) regression. After preprocessing, time series of 112 brain areas (regions of interest [ROIs]) delineated by Harvard Oxford atlas (Makris et al., 1999) were extracted. FC was calculated by computing Pearson correlation coefficients of the time series between each pair of 112 ROIs, and then Fisher’s r-to-z transform was performed to attain normalized z scores.

**MDD subtyping and validation analysis**

Consensus clustering, an approach to detect communities in complex networks (Rasero, Diez, Cortes, Marinazzo, & Stramaglia, 2019; Rasero et al., 2017), was applied to characterize MDD subtypes. Age, sex, education, and FD value were regressed out from harmonized functional connectivity (FC) matrices (constructed by using both positive and negative FC) before clustering. Then the distance between MDD patients *u* and *v* ($d_{uv}^{i}$) were calculated based on the Euclidean distances of FC between each ROI (*i*) and any other ROIs in the network:

$$D^{i}\equiv d_{uv}^{i}=\sqrt{\sum_{j=1}^{N} {({FC}_{ij}^{u}-{FC}_{ij}^{v})}^{2}}$$

where *D^i^* denotes the distance matrix between each participant, *i*$\neq$*j*, and *N* is the total number of ROIs (here is 112 in our study). Then, each distance matrix *D^i^* was partitioned into *k* clusters using *k*-medoids method which is more robust to noise and outliers than *k*-means (Kaufman & Rousseeuw, 2009), and the results of clustering would be encoded into a binary adjacency matrix. One indicated the corresponding pair of participants belonged to the same cluster and zero otherwise. By averaging binary adjacency matrices of all ROIs, the consensus matrix *C* for the current *k* can be obtained. The *k* value corresponds to a different scale or resolution of clustering, that is, larger *k* results in more and smaller clusters, meaning higher resolution but less overall information. According to the optimizing strategy for capturing both large-scale (small *k* values) and small-scale (large *k* values) structural details, a final consensus matrix was evaluated by averaging all *C* over the *k* range in the interval (2-20 here as other studies used (Rasero et al., 2023)) (Rasero et al., 2017). The final consensus matrix *C* was further partitioned into communities by Newman and Girvan-like modularity maximization (Newman & Girvan, 2004). In detail, firstly the modularity matrix *B* was constructed:

$$B=C-P$$

where *P* is the expected co-assignment matrix, uniform as a consequence of the null ensemble, obtained by randomly permuting the cluster labels found by *k*-medoids method. Modularity matrix *B* was further fed into a generalized Louvain algorithm (<https://github.com/GenLouvain/GenLouvain>) to obtain an optimal output partition (i.e., FC-based MDD subtypes for the present study) that maximized the network modularity. Bootstrapping was applied to assess the stability of each subtype and the 95% confidence intervals (CIs) of the estimated maximum modularity which reflects the statistical difference from zero.

To validate our subtyping results in varied aspects, 1) we replicated the consensus clustering analysis with Power atlas (Power et al., 2011), which offers a higher resolution with 264 ROIs. The similarity between the clusters derived from Power atlas and those from our primary analysis was evaluated by normalized mutual information (NMI) (Rasero et al., 2023). The range of NMI is from zero to one, with a larger value indicating a greater similarity.

2) Further, given the implicit resolution dependence when clustering of the modularity matrix *B* (Reichardt & Bornholdt, 2006), a multi-resolution hierarchical clustering, which obtains community structure after sampling the entire range of possible resolutions (Jeub, Sporns, & Fortunato, 2018), was used to validate the stably acquired clusters from consensus clustering. The similarity between the major subtypes obtained from hierarchical clustering and consensus clustering was evaluated by NMI.

3) To further validate the robustness of subtyping solutions, an additional validation approach was conducted using a cross-validation. Specifically, the original dataset was divided into two halves using stratified random sampling, and the full MDD dataset was stratified based on age, sex, education level, and mean framewise displacement (FD) to ensure an even distribution of these covariates across the two halves. This stratification allowed us to reduce potential sampling bias and maintain demographic and head-motion balance between the training and test sets. Both subsamples subsequently underwent data harmonization and covariate regression with adjustments for age, sex, education, and head motion, identical to the main analysis. After that, we replicated the consensus clustering analysis in each subsample. For quantitative assessment of subtype generalizability, a support vector machine classifier was trained on the first half with whole-brain FC features and observed labels provided by its subtyping solution. A grid search procedure with ten-fold cross-validation was employed to optimize hyperparameters. The optimized classifier was then applied to predict the subtype label for the two major subtypes in the second subsample. Predictive validity was evaluated by calculating classification accuracy between predicted subtype labels and those observed labels from the clustering analysis of the second subsample.

4) To ensure that the identified subtypes were not influenced by medication effects (Yan et al., 2019), we conducted consensus clustering specifically within first-episode drug-naïve patients with MDD and evaluated the similarity between clusters derived from drug-naïve patients in the validation analysis and all patients in the main analysis using NMI.

**Clinical and FC characteristics of MDD subtypes**

After we found robust FC-based MDD subtypes, the differences in age, education, illness duration, HAMD and HAMA total scores between MDD subtypes (two subtypes identified in the present study, see results) were tested by two-sample t-test, while sex, episode status, and medication by chi-square test. Multiple linear regression was applied to assess the differences in the overall connectivity per participant between each subtype and HCs group with age, sex, education, and FD value as covariates. The overall connectivity for each participant was defined as the average value of both positive and negative FC of the above-mentioned harmonized connectivity matrix in the main analysis.

To further evaluate the differences in overall connectivity, we also set the median and 10% trimmed mean of all FC (i.e., excluding the 10% largest and 10% smallest values and then calculating the mean of the remaining 80% values), average positive FC, and average of absolute values of all FC as the overall connectivity metrics and then repeated above between-group comparisons. A significance *P*-value of 0.05 was set for all the clinical and overall connectivity comparisons.

**Epicenter identification**

After subtyping, in order to identify the epicenter in each subtype group derived from the FC alternations, multivariate distance matrix regression (MDMR) (Shehzad et al., 2014) was first applied to calculate the FC alterations in all brain regions between each MDD subtype and HCs. For a certain ROI (*i*), MDMR was used to assess the extent to which the group factor (MDD subtype vs. HCs) can explain the FC dissimilarity between pairs of participants within these two groups. This was achieved by regressing the distance matrix onto a set of predictors and further computing a pseudo-R^2^ effect size:

$$R_{x}^{2}=\frac{tr(H_{x}G^{i})}{tr(G^{i})}$$

where *tr* denotes the trace operator, *Hx* is the isolated effect of predictor *x* with $H=X{(X^{T}X)}^{-1}X^{T}$, *G^i^* is the so-called Gower matrix constructed by the distance matrix*.* The predictors in the model included the present group factor (MDD subtype vs. HCs) with covariates (including age, sex, education, and FD value) to mitigate their influences. Similar to ordinary R^2^ statistics in an ANOVA analysis, the value of pseudo-R^2^ ranges from 0 to 1, where a higher pseudo-R^2^ value indicates greater FC differences in this ROI between groups and suggests greater FC alterations in an MDD subtype relative to HCs in the present study. After calculating pseudo-R^2^ in every ROI within both MDD subtypes, we attained pseudo-R^2^ maps for each subtype group which will be used for subsequent identification of epicenter and for correlation analyses between FC alterations and transcriptomics, neurotransmitter receptor/transporter, and cognition.

We used the method of epicenters identification for psychiatric disorders as a prior work did (Shafiei et al., 2020). Among brain regions of individuals within the same subtype, a region with a larger FC alteration (larger Pseudo-R^2^, here) compared to other regions could potentially be an epicenter of this subtype if its neighbors also have higher FC alterations. The neighbors of *i*-th brain region were the regions that are connected to node *i* by FC. The collective information of the neighbors (*D*_i_) was evaluated by weighted mean FC alterations of the neighbors:

$$D_{i}=\frac{1}{N_{i}}\sum_{j\neq i, j=1}^{N_{i}} d_{j}*{FC}_{ij}$$

where *N*_i_ is the total number of regions that are connected to node *i* (here *N*_i_ is 111 as the fully connected network we used), *d* is the Pseudo-R^2^, and *FC*_ij_ is the mean FC value (including both positive and negative FC) between nodes *i* and *j* across individuals of each subtype. Nodes were ranked based on their Pseudo-R^2^ and their neighbors’ collective information in ascending order in two separate lists, and we then identified nodes that were highly ranked in both lists and assessed the significance of rankings using the spatial permutation testing (spin tests; 1000 repetitions with *P*<0.05 as a significance).

**Biological characteristics of MDD subtypes**

*Transcriptomics*

Brain gene transcription data were obtained from the public Allen Human Brain Atlas (AHBA) (Hawrylycz et al., 2012). The dataset was derived from six healthy postmortem adult brains, including more than 20000 gene expression data at 3702 brain regions. We extracted the gene expression data to the 112 ROIs of Harvard Oxford template using abagen toolbox (Ross et al., 2021), with a proposed preprocessing pipeline (Arnatkeviciute, Fulcher, & Fornito, 2019). In short, the primary steps included: 1) probe re-annotation; 2) intensity-based filtering of probes to exclude those that do not exceed background noise in at least 50% of samples; 3) selection of presentative probe with differential stability method; 4) assigning samples to brain regions from Harvard atlas; 5) normalizing the expression data with scaled robust sigmoid method. After preprocessing, there were 15633 genes survived. As AHBA includes genes expressed in any body tissue, we focused on the 1920 brain-specific genes relative to other tissues extracted from the Human Protein Atlas (<https://www.proteinatlas.org>). The precise list of 1920 brain-specific genes is provided in Table S5.

*Associations between FC alterations and transcriptomics*

As all donors provided sampling sites of genes in left hemisphere, but only two of six donors from the AHBA dataset were sampled in both left and right hemispheres, we restricted our association analysis between gene expressions and FC alterations (i.e., pseudo-R^2^ map) in the 56 ROIs to the left hemisphere data. Partial least squares (PLS) regression was used to estimate the association between FC alterations in each MDD subtype relative to HCs and the gene expression measurements for 1920 brain-specific genes. The first PLS component (PLS1) is the linear combination of the weighted gene expression score that is not only most strongly correlated with the FC alteration map but also explains the largest portion of variance in FC alterations (approximately 28.5% in our study) among all PLS components. Given potential inflation of false positives due to spatial autocorrelation in the gene expression data, we followed recommendations by Fulcher et al. (Fulcher, Arnatkeviciute, & Fornito, 2021). Specifically, the statistical significance of PLS1 was tested by permuting the response variables (FC alterations map, here) instead of genetic expression matrix 1000 times to build a null distribution while keeping gene expression matrix unchanged. The *P* value was estimated as the percentage of null correlations that exceeded the primary correlations between PLS1 and FC alterations map estimated on the original data. The estimation error of each gene’s PLS1 weight was assessed by bootstrapping (resampling with replacement of 56 brain regions in the left hemisphere), and the ratio of the weight of each gene to its bootstrap standard error was used to calculate the Z scores that reflect the gene’s contribution to PLS1 (Whitaker et al., 2016). Then we can identify the significant genes by calculating the *P* value from Z score. The genes with PLS1 weights *P_FDR_*<0.05 were set as significant. Further, these significant genes in each subtype were divided into two groups (PLS1+ and PLS1-) according to their positive or negative correlation coefficients, which were used for subsequent analyses.

To gain deeper insights into the functional roles and interactions of significant genes found in PLS1, enrichment analyses were employed. In the present study, gene ontology (GO) biological process enrichment analyses and identification of hub genes were performed separately for significant genes in PLS1+ and PLS1- in each subtype. Specifically, GO enrichment analysis offers a systematic approach to categorize genes into hierarchical classes based on their biological processes (Gaudet et al., 2021), thus we can infer the predominant biological activities and pathways perturbed in MDD. Further, via mapping the physical and functional interactions between the proteins encoded by the significant genes, protein-protein interaction (PPI) networks allow us to identify hub genes, which are highly connected nodes within the network and often play pivotal roles in maintaining cellular functions and regulatory mechanisms. Hub genes are also frequently implicated in disease processes, possibly serving as potential biomarkers or therapeutic targets (Martino, Chiarugi, Margheriti, & Garau, 2021). In a word, the integration of GO enrichment analysis and PPI network construction provides a holistic view of the molecular underpinnings of MDD pathogenesis. In our study, GO biological process enrichment analysis was conducted by using Metascape toolbox ([https://metascape.org/gp/index.html#/main/step1](https://metascape.org/gp/index.html#/main/step1)) (Zhou et al., 2019) with *P_FDR_*<0.05 set as significant, and the top ten significant GO terms were reported here. Next, to identify the hub genes, we first constructed the PPI networks with the significant genes using String version 12.0 (<https://string-db.org/>). Then, Cytoscape version 3.10.0 plugin *cytoHubba* (<https://cytoscape.org/>) was used to analyze the constructed PPI networks based on the maximal clique centrality (MCC) algorithm, where MCC value reflects network centrality for each gene. MCC algorithm offers a more comprehensive measure of node centrality compared to other approaches for considering the node's involvement in fully connected subgraphs (Chin et al., 2014). This approach captures the structural and topological significance of nodes within the network, providing a robust identification of hub genes. Based on this, genes with the top three highest MCC values in each constructed network were selected as hub genes and reported here.

*Associations between FC alterations and cognition*

We also explored the association between FC alterations of each MDD subtype and cognitive domains. The brain maps of meta-analytic cognitive terms were extracted from Neurosynth (<http://neurosynth.org/>). We selected 124 cognitive terms that existed in both of Neurosynth and Cognitive atlas databases (<http://cognitiveatlas.org/>) (Yarkoni, Poldrack, Nichols, Van Essen, & Wager, 2011) as a prior work did (Wei et al., 2023) (Table S8), and mapped those brain maps of selected cognitive terms onto the Harvard atlas for subsequent association analysis. The relationship between FC alterations in each MDD subtype and cognitions was evaluated by Pearson correlation with spin permutations test (1000 times with *P_FDR_*<0.05 as significant).

**Supplementary results**

**Validation for MDD subtypes**

To evaluate cross-atlas generalizability, we replicated the analysis using Power-264 atlas, which consistently identified two major stable MDD subtypes (modularity=0.11, 95% CI 0.101-0.119; stability score: 0.969 and 0.75, respectively). While some atlas-related variations were expected, the subtypes derived from Power-264 atlas showed quantifiable spatial correspondence (NMI=0.6) with those derived from Harvard-Oxford atlas, collectively supporting the robustness of our findings.

Regarding varied clustering methods, robustness analyses revealed that there’s a large similarity (NMI=0.732) between the two major subtypes obtained by multi-resolution hierarchical clustering (Figure S2) and consensus clustering.

In the machine learning cross-validation analysis, the first half subsample identified two major MDD subtypes using consensus clustering (269 and 158 patients, respectively), and the second half also yielded two subtypes of comparable patients (279 and 119, respectively). The predictive accuracy between predicted and observed labels was 95.7%, showing a high agreement between the two subsamples, and thus confirming the reliability of our subtyping results across random data partitions.

In analyses restricted to the first-episode drug-naïve patients with MDD, we also observed significant modularity (0.185, 95% CI 0.165-0.206) and high stability scores of 0.989 and 0.972 for each subtype. These findings provide robust evidence for the stable existence of MDD subtypes independent of medication confounders. Further, a high similarity was observed between subtypes derived from drug-naïve patients and the full sample (NMI=0.77).

**Associations between FC alterations and transcriptomics**

The FC alteration patterns in both MDD subtypes were associated with brain gene expression profiles (subtype 1, *r*=0.40, *P_perm_*=0.002; subtype 2, *r*=0.49, *P_perm_*=0.001). Based on this, significant genes were further identified and further used for enrichment analysis. For subtype 1, the top ten significant GO biological process enrichment of genes with *positive* weights included inorganic ion transmembrane transport, regulation of presynaptic membrane potential, chemical synaptic transmission, neuromuscular process, synapse organization, axon development, neuronal action potential propagation, regulation of monoatomic ion transport, positive regulation of developmental growth, and regulation of postsynaptic membrane potential (Figure 3B). The top ten significant GO biological process enrichment of genes with *negative* weights included regulation of trans-synaptic signaling, synaptic signaling, synapse organization, brain development, inorganic ion transmembrane transport, glial cell differentiation, regulation of plasma membrane bounded cell projection organization, memory, positive regulation of neuroblast proliferation, and neuron projection development (Figure 3C) .

For subtype 2, the top ten significant GO biological process enrichment of genes with *positive* weights included synapse organization, chemical synaptic transmission, axon development, regulation of neuron differentiation, neural nucleus development, synaptic vesicle cycle, response to metal ion, positive regulation of developmental growth, glucose transmembrane transport, and sodium ion transport (Figure 3E). The top ten significant GO biological process enrichment of genes with *negative* weights included modulation of chemical synaptic transmission, trans-synaptic signaling, regulation of cell projection organization, regulation of nervous system development, brain development, regulation of neuronal synaptic plasticity, neuron projection development, receptor-mediated endocytosis, adenylate cyclase-inhibiting G protein-coupled receptor signaling pathway, and inorganic ion transmembrane transport (Figure 3F).

For subtype 1, the hub genes of the PLS1+ genes constructed PPI network were SCN1A, KCNAB3, and KCNA1 (all MCC values=8), while the hub genes of the PLS1- genes were GABBR1, GABRB1, and GABRG1 (MCC value=13, 12, 12, respectively) (Table S6). For subtype 2, the hub genes of the PLS1+ genes constructed PPI network were SCN1A, SCN1B, KCNA1 (MCC value=9, 8, 8, respectively), while the hub genes of the PLS1- genes were DLG4, GRIA1, and GRIN2B (MCC value=26, 24, 22, respectively) (Table S6). The shared hub genes’ function in both subtypes was related to membrane potential and neurotransmitter release. The genes related to GABA receptor were specifically found in subtype 1, while genes related to glutamate receptor in subtype 2.

**Associations between FC alterations and cognition**

Both subtypes showed associations between FC alterations and meta-analysis-based maps of cognitive terms related to speech perception (subtype 1, *r*=0.33, *P_FDR_*=0.031; subtype 2, *r*=0.43, *P_FDR_*=0.048), categorization (subtype 1, *r*=0.35, *P_FDR_*=0.031; subtype 2, *r*=0.48, *P_FDR_*=0.048), localization (subtype 1, *r*=0.39, *P_FDR_*=0.031; subtype 2, *r*=0.50, *P_FDR_*=0.048), and multisensory (subtype 1, *r*=0.42, *P_FDR_*=0.031; subtype 2, *r*=0.45, *P_FDR_*=0.048). For subtype 2, cognitive terms including integration (*r*=0.44, *P_FDR_*=0.048), adaptation (*r*=0.32, *P_FDR_*=0.048), perception (*r*=0.39, *P_FDR_*=0.048), expertise (*r*=0.29, *P_FDR_*=0.048), detection (*r*=0.41, *P_FDR_*=0.048), discrimination (*r*=0.38, *P_FDR_*=0.048), speech production (*r*=0.42, *P_FDR_*=0.048), selective attention (*r*=0.44, *P_FDR_*=0.048), and priming (*r*=0.32, *P_FDR_*=0.048) were spatially correlated with the FC alterations (Table S8 and Figure S3).

To better interpret these findings, we further adopted the classification approach of the cognitive atlas database (<http://cognitiveatlas.org/>) to summarize these cognitive terms. Specifically, the shared cognitive classifications for both subtypes were language, reasoning and decision making, perception, and multisensory; whereas no classification were specific to subtype 1 but learning and memory, and attention were specific to subtype 2 (Table S9).

**Table S1.** Demographic characteristics for participants in each institution included in our study.

| Site Index^a^ | Whole (MDD and HC) | | |  | MDD | | |  | HC | | |
| --- | --- | --- | --- | --- | --- | --- | --- | --- | --- | --- | --- |
|  | No. (Female/male) | Age | Education |  | No. (Female/male) | Age | Education |  | No. (Female/male) | Age | Education |
| 1 | 146(84/62) | 31.8±8.5 | 14.5±2.7 |  | 73(43/30） | 31.9±8.1 | 13.8±3 |  | 73(41/32) | 31.7±9 | 15.2±2.3 |
| 2 | 30(25/5) | 43.5±11.7 | 10.8±4.6 |  | 16(15/1） | 41.8±11.5 | 11.6±4.5 |  | 14(10/4) | 45.6±12.1 | 10±4.8 |
| 7 | 72(45/27) | 40±11.8 | 13.1±4.5 |  | 35(22/13） | 41.9±11.7 | 11.1±4 |  | 37(23/14) | 38.2±11.8 | 14.9±4.1 |
| 8 | 114(71/43) | 31.5±10.1 | 12.3±3 |  | 53(35/18） | 31.8±9.5 | 11.3±3.2 |  | 61(36/25) | 31.2±10.7 | 13.1±2.5 |
| 9 | 96(44/52) | 28.6±8.3 | 14.7±3.1 |  | 48(26/22） | 28.6±8.7 | 13.4±2.9 |  | 48(18/30) | 28.6±8 | 15.9±2.8 |
| 10 | 71(33/38) | 32.7±9.8 | 11.9±2.8 |  | 45(24/21） | 32.7±10.8 | 11.3±3.1 |  | 26(9/17) | 32.7±8.1 | 12.8±2 |
| 11 | 37(20/17) | 30.8±9.3 | 13.2±3.6 |  | 20(11/9） | 30.2±9.3 | 11.2±3 |  | 17(9/8) | 31.4±9.6 | 15.6±2.5 |
| 13 | 36(23/13) | 33.4±9.5 | 13.5±2.2 |  | 20(12/8） | 32.7±8.6 | 13.7±2.2 |  | 16(11/5) | 34.4±10.7 | 13.3±2.3 |
| 14 | 93(59/34) | 29.9±6.3 | 14±3.2 |  | 61(42/19） | 30.1±7 | 13.7±3.3 |  | 32(17/15) | 29.6±5 | 14.6±2.8 |
| 15 | 67(41/26) | 42.8±14.1 | 12.2±3.9 |  | 30(21/9） | 46.5±12.6 | 11.1±3.8 |  | 37(20/17) | 39.8±14.7 | 13.1±3.8 |
| 17 | 82(55/27) | 21.2±2.5 | 13.5±1.5 |  | 41(27/14） | 21.7±3 | 13.1±1.5 |  | 41(28/13) | 20.6±1.8 | 13.8±1.6 |
| 19 | 49(30/19) | 35.1±10.6 | 9.8±3.6 |  | 18(13/5） | 34.9±11.4 | 9.7±3.1 |  | 31(17/14) | 35.2±10.2 | 9.9±3.9 |
| 20 | 479(322/157) | 39±13.9 | 12±3.7 |  | 250(166/84） | 38.5±11.9 | 10.9±3.4 |  | 229(156/73) | 39.6±15.7 | 13±3.8 |
| 21 | 144(82/62) | 35.2±12.3 | 12.3±2.5 |  | 79(45/34） | 34.1±12.1 | 11.8±2.7 |  | 65(37/28) | 36.5±12.5 | 13±2.1 |
| 22 | 38(17/21) | 28.8±9.6 | 12.7±2.6 |  | 18(9/9） | 33.8±9.8 | 12±3 |  | 20(8/12) | 24.4±7.1 | 13.3±2.1 |
| 23 | 45(27/18) | 29.7±10.5 | 14.1±3.6 |  | 22(12/10） | 26.2±7.4 | 13.9±3.2 |  | 23(15/8) | 33±12 | 14.3±4.1 |
| Total | 1599(978/621) | 34.5±12.4 | 12.7±3.5 |  | 829(523/306） | 34.4±11.6 | 11.9±3.4 |  | 770(455/315) | 34.6±13.2 | 13.5±3.4 |

^a^ The code of site index is consistent with the study of Yan et.al.(Yan et al., 2019), and the name of corresponding sites can be found in Table S3 of that reference.

Abbreviation: MDD, major depressive disorder; HC, healthy controls.

**Table S2.** Demographic and clinical characteristics of all participants included in our study.

| Variable | MDD | HC | *P* value^d^ |
| --- | --- | --- | --- |
| Sample size, N | 829 | 770 | - |
| Age, years, mean±SD | 34.4±11.6 | 34.6±13.2 | 0.775 |
| Sex, N, Female/male | 523/306 | 455/315 | 0.101 |
| education, years, mean±SD | 11.9±3.4 | 13.5±3.4 | **<0.001** |
| Head motion (FD), mm, mean±SD | 0.068±0.036 | 0.069±0.037 | 0.524 |
| Illness duration^a^, months, mean±SD | 39.1±61.1 | - | - |
| HAMD total score^b^, mean±SD | 21.3±6.6 | - | - |
| HAMA total score^c^, mean±SD | 19.2±9.1 | - | - |
| Episode status |  | - | - |
| First-episode, N(%) | 417(50.3%) | - | - |
| Recurrent, N(%) | 208(25.1%) | - | - |
| Unknown, N(%) | 204(24.6%) | - | - |
| Medication status |  | - | - |
| Drug-naive, N(%) | 313(37.8%) | - | - |
| On medication, N(%) | 222(26.8%) | - | - |
| Unknown, N(%) | 294(35.5%) | - | - |

^a^ Data were available for 697 of 829 patients

^b^ Data were available for 745 of 829 patients

^c^ Data were available for 522 of 829 patients

^d^ *P* value was calculated by two-sample t-test (age, education) and chi-square test (sex)

Abbreviation: MDD, major depressive disorder; HC, healthy controls; FD, Framewise displacement; HAMD, Hamilton Depression rating scale; HAMA, Hamilton Anxiety rating scale.

**Table S3.** MRI scanning parameters for each site

| Site Index^a^ | Scanner | Head coil (channel) | TR (s) | TE (ms) | Flip angle (°) | Thickness/gap (mm) | No. axial slices | No. volumes | Voxel size (mm3) | FOV |
| --- | --- | --- | --- | --- | --- | --- | --- | --- | --- | --- |
| 1 | Siemens Tim Trio 3T | 32 | 2 | 30 | 90 | 4/0.8 | 30 | 210 | 3.28×3.28×4.8 | 210×210 |
| 2 | Philips Achieva 3T | 8 | 2 | 30 | 90 | 4/0 | 37 | 200 | 1.67×1.67×4 | 240×240 |
| 7 | GE Discovery MR750 3T | 8 | 2 | 30 | 90 | 3.2/0 | 37 | 184 | 2.29×2.29×3.2 | 220×220 |
| 8 | GE Sigma 3T | 8 | 2 | 30 | 90 | 3/0 | 35 | 200 | 3.75×3.75×3 | 240×240 |
| 9 | GE Discovery MR750 3T | 8 | 2 | 25 | 90 | 3/1 | 35 | 200 | 3.75×3.75×4 | 240×240 |
| 10 | Siemens Tim Trio 3T | 32 | 2 | 30 | 90 | 3/1.52 | 32 | 212 | 3.75×3.75×4.52 | 240×240 |
| 11 | GE Sigma 3T | 8 | 2 | 30 | 90 | 5/- | 33 | 200 | 3.75×3.75×5 | 240×240 |
| 13 | GE Excite 1.5T | 16 | 2.5 | 35 | 90 | 4/0 | 36 | 150 | 4×4×4 | 256×256 |
| 14 | Siemens Tim Trio 3T | 32 | 2.5 | 25 | 90 | 3.5/0 | 39 | 200 | 3.75×3.75×3.5 | 240×240 |
| 15 | Siemens Verio 3T | 12 | 2 | 25 | 90 | 4/0 | 36 | 240 | 3.75×3.75×4 | 240×240 |
| 17 | GE Sigma 3T | 8 | 2 | 40 | 90 | 4/0 | 33 | 240 | 3.75×3.75×4 | 240×240 |
| 19 | GE Sigma 3T | 8 | 2 | 22.5 | 30 | 4/0.6 | 33 | 240 | 3.44×3.44×4.6 | 220×220 |
| 20 | Siemens Tim Trio 3T | 12 | 2 | 30 | 90 | 3/1 | 32 | 242 | 3.44×3.44×4 | 220×220 |
| 21 | Siemens Tim Trio 3T | 32 | 2 | 30 | 90 | 3.5/0.7 | 33 | 240 | 3.12×3.12×4.2 | 200×200 |
| 22 | Philips Gyroscan Achieva 3T | 32 | 2 | 30 | 90 | 4/0 | 36 | 250 | 1.67×1.67×4 | 240×240 |
| 23 | Philips Achieva 3T | 8 | 2 | 30 | 90 | 4/0 | 38 | 240 | 3.75×3.75×4 | 240×240 |

^a^ The code of site index is consistent with the study of Yan et.al.(Yan et al., 2019), and the name of corresponding sites can be found in Table S3 of that reference.

Abbreviation: TR, repetition time; TE, echo time; No., number; FOV, field of view.

**Table S4.** The differences in overall functional connectivity between the whole each patient subtype and controls, respectively, which was calculated by different definitions to validate the main results.

| Metrics | Subtype 1 vs. HC | | |  | Subtype 2 vs. HC | | |  | Whole MDD vs. Hc | | |
| --- | --- | --- | --- | --- | --- | --- | --- | --- | --- | --- | --- |
|  | *β* | *t*_1291_ | *P* |  | *β* | *t*_1063_ | *P* |  | *β* | *t*_1593_ | *P* |
| mean of pos FC | -0.067 | -16.510 | **<0.001** |  | 0.082 | 13.560 | **<0.001** |  | -0.0137 | -2.7 | **0.007** |
| median of all FC | -0.088 | -16.922 | **<0.001** |  | 0.104 | 14.147 | **<0.001** |  | -0.0176 | -3.099 | **0.002** |
| trimmed mean of all FC | -0.087 | -16.970 | **<0.001** |  | 0.102 | 14.138 | **<0.001** |  | -0.013 | -2.672 | **0.008** |
| mean of absolute value of all FC | -0.073 | -16.801 | **<0.001** |  | 0.089 | 13.812 | **<0.001** |  | -0.0139 | -2.777 | **0.006** |

Abbreviation: FC, functional connectivity; HC, healthy controls; pos, positive; neg, negative; MDD, major depressive disorder.

**Table S5.** The list of brain-specific genes.

| **Gene label** | | | | | | | | | |
| --- | --- | --- | --- | --- | --- | --- | --- | --- | --- |
| ADAM22 | ADRA1D | LDLRAP1 | SLC18A2 | PSD2 | ST8SIA3 | MPZ | S100B | ZCCHC18 | PCDHGC3 |
| SCN1B | TMEM229B | LINC00672 | PABPC1L2B | GDAP1 | BCL11B | NPTX1 | MID1IP1 | WDR17 | GPC5 |
| KCNC3 | SV2C | BRSK1 | MAGEE1 | PTER | PLXNB3 | LRFN4 | ADAP1 | HTR2C | RGS20 |
| PELI3 | FGF17 | BRICD5 | LRRC8B | SHISA6 | NYAP1 | SOGA1 | JAKMIP1 | ACTR3B | CELF3 |
| SEMA7A | VWA5B2 | CCDC92 | PPP1R14A | PCP4 | HCRT | PRKAR1B | HEPACAM | SLC9A7 | C3orf14 |
| SOHLH1 | TSPAN5 | LRRC75B | NRIP3 | POU3F3 | NRG3 | AJAP1 | BAALC | SYNGR3 | CLEC9A |
| NKX6-3 | CACNG8 | PIEZO2 | NKX6-1 | ZNF337 | RAB3A | AJM1 | NTRK3 | CALB2 | MDGA1 |
| UBE2QL1 | CRYGS | P2RX7 | RAB26 | IGSF8 | PAIP2B | GAD2 | PDYN | MYT1 | IGSF22 |
| KCNS1 | IQSEC3 | GNAI1 | TMEM229A | ANKRD40 | LMO4 | RIMS2 | FRRS1L | LINGO3 | WNT10B |
| MAPK10 | OTUD7A | DGKD | TMEM35A | CACNA1B | PCDH8 | MFSD13A | RFX4 | TBCB | APOE |
| FGF9 | KLHL3 | DIRAS2 | NKX2-8 | C1orf216 | KIF5C | RIT2 | SAPCD2 | NSMF | CALB1 |
| RNF157 | PRKCE | GPR83 | NRSN1 | NECAP1 | CLEC4G | ARL9 | HAP1 | LRRC7 | KLRC2 |
| EIF4E1B | INPP5F | ZNF30 | CNTNAP5 | S1PR5 | FIBCD1 | PHLPP1 | RGS1 | BTBD17 | NSG2 |
| HAPLN4 | DLGAP1 | AKAP6 | ENO2 | FMNL2 | CNTN6 | ENC1 | CYFIP2 | ESPNL | BCAN |
| BEND6 | SPTBN4 | ELMO1 | UNC79 | SSTR3 | DUSP8 | SLC25A53 | VSTM2B | AGAP2 | NNAT |
| KCNC1 | GPR22 | FAM221A | FA2H | TMEM235 | AZIN2 | SYT5 | ATP1B2 | ROBO2 | PEA15 |
| EMX1 | DTNB | CLSTN3 | ACYP2 | PDE10A | PGBD5 | CACNG3 | RIMS1 | RGS9 | CNMD |
| CADPS2 | TMOD2 | ARHGAP44 | SLITRK5 | FEZ1 | SLC22A31 | RTN4R | LHX8 | CDH4 | MANEAL |
| RHBDL3 | CELSR3 | CSMD1 | CCDC78 | PPP2R5B | CCK | FAM131C | EFR3B | ZSWIM9 | CHST1 |
| SYNDIG1 | NEUROD1 | DOHH | FEZF2 | PLLP | MGAT5B | SERTM1 | FAM184B | PRRG3 | C1QL1 |
| KCNA1 | C2CD4D | PRRG1 | MFSD4A | NSG1 | MPP2 | KIF6 | CENPB | GRIK2 | FBXO2 |
| TMEM81 | ITPK1 | RASD2 | ADCY2 | SUSD4 | ADGRB2 | PITX3 | HPDL | GRIN3A | GABRA2 |
| SORT1 | CBLN4 | B4GALNT1 | FAM171A1 | RTP1 | RND2 | PPP1R17 | SYNDIG1L | STMN3 | TMEM132A |
| SCRT1 | YPEL4 | SLC17A6 | NANOS3 | TMEM163 | SEC61A2 | LMTK3 | PRKCZ | CSMD3 | FAM107A |
| CKMT1B | NRN1 | ELAVL2 | KCNK1 | HS3ST4 | NBEA | MISP3 | PPP1R1B | APLNR | CX3CR1 |
| ADAM23 | SLCO3A1 | GDPD1 | BHLHE22 | CCSAP | LPAR1 | ARHGEF4 | CNRIP1 | SOWAHA | DNAJC12 |
| JAKMIP3 | DTX4 | MCHR2 | R3HDM1 | RAB39B | TLL2 | PPEF1 | SPTBN2 | NLGN3 | NOL4 |
| PKD1 | MAP6D1 | CNDP1 | SYT11 | CDH22 | FGF13 | EID2B | PMP2 | FAM167A | AQP4 |
| ST8SIA5 | PLCB1 | PLEKHH1 | SLAIN1 | GPR62 | GPRASP1 | ASIC4 | NR2E1 | B4GAT1 | ATAT1 |
| GRAMD1B | CACNA1A | PCSK6 | DGKG | DNM1 | MAPK11 | SOX8 | FZD9 | PNCK | PLD6 |
| NREP | KIF25 | PXK | DTNA | PPFIA2 | ADCY8 | DAB1 | MYH15 | FAM181B | NCAN |
| KCNS2 | PSRC1 | UCN | CMTM5 | HTR4 | SOX10 | SEZ6L | SLIT1 | TAGLN3 | CELF5 |
| RGS6 | TRIM3 | FRMPD4 | TMEM178B | C10orf90 | CDYL2 | CHST6 | ANO3 | ADGRB3 | PAX6 |
| CHRD | PPM1H | RHBDL1 | EPHA4 | PCDHGA7 | PCDHA1 | GRM4 | DLG4 | GABRE | CRMP1 |
| ARHGAP39 | DND1 | HAPLN2 | FRS3 | CADM4 | PAQR6 | ICAM5 | SYTL5 | FBXO41 | WNT7B |
| SEC14L5 | MAP7D2 | NEFL | NRG2 | CTNNA3 | SRCIN1 | SPEF1 | GRIN2B | LILRB4 | CPT1C |
| KCNC4 | MCF2 | BEAN1 | AIFM3 | IL1RAPL1 | SIAH3 | ITPKB | RAB11FIP4 | RANBP3L | MSANTD1 |
| ABCC8 | SLC12A5 | SP9 | MOG | STMN2 | RGS14 | PRMT8 | KCTD17 | SORCS3 | XKR4 |
| STAC2 | C3orf18 | TFAP2E | NAP1L5 | WASF1 | RASL10B | CCDC85A | DYNC1I1 | PLPPR1 | NTSR2 |
| RAPGEF5 | ANKRD24 | POU3F2 | NTRK2 | AGPAT4 | TRIM17 | AP1S1 | LRP4 | PRKCG | IQCA1 |
| VAMP1 | EDIL3 | ABCA2 | CAPN3 | RAB15 | GPR176 | FGF1 | USP27X | PNOC | GPR27 |
| CABP1 | GPHN | PRAG1 | MAPRE3 | GRIN2D | PPP2R2C | GRM1 | MAPK8IP1 | HBQ1 | GPM6B |
| SLC4A8 | VSNL1 | SLC9A5 | BSCL2 | SCG5 | MOB3B | ATP1A2 | GNG3 | TENM1 | CACNG4 |
| TNFRSF25 | SPTBN5 | PCP4L1 | RFPL1 | SCRN1 | VWC2L | CAMK2N2 | NDRG2 | SMOC1 | KIF21B |
| SCN1A | CCER2 | CCNE2 | PLP1 | PDE1B | GPR12 | MAST3 | DBX2 | STX1A | P2RY12 |
| SCX | CACNA2D3 | RNFT2 | ZNF382 | P2RX2 | HTR3B | KIF19 | OPRK1 | CLDN10 | SST |
| ZFPM2 | COL19A1 | CYP26B1 | SYN1 | KCND1 | OPN4 | CRHBP | SCG2 | C1orf122 | DLG2 |
| ACSL6 | RNF208 | NIPAL3 | PRR3 | CCNB3 | ERICH3 | CECR2 | TSC22D4 | SHISA7 | TSPOAP1 |
| KCNA2 | PLEKHM2 | RNF112 | PCDHB9 | ABHD17A | MAP4K4 | C1orf198 | RTP5 | KCNQ2 | DOK6 |
| NAT8L | CAMKK1 | GPR85 | PCBP4 | ENTPD3 | SLCO1A2 | C4orf48 | OLIG1 | IL17RB | CDKN2D |
| DIRAS1 | EPHB6 | TMEM120B | TMEM132D | GNG7 | HMX2 | HPSE2 | KLF16 | GRM3 | TTYH1 |
| TLE2 | CCDC88A | SGTB | AMER3 | PLCXD3 | PKP4 | GNAL | GPR173 | SMIM10L2B | ETNPPL |
| POU6F2 | RUFY2 | TMEM132E | NLGN4X | SHTN1 | GNAZ | ADRA1B | RAB3C | PRODH | SCN3B |
| SNAP25 | CNKSR2 | ADGRA1 | ETV1 | FAM43B | SCGN | SCD | GSG1L | SHANK1 | LIN7B |
| TIGD3 | MAP7 | TMEM63A | PENK | LMAN1L | TUBB4A | ARNT2 | WASF3 | OPRM1 | CPNE6 |
| OR2L13 | TSPAN9 | PAGR1 | ACTL6B | TMC6 | BSN | TUBB3 | SYT17 | GPR37L1 | PTPRZ1 |
| MTCL1 | SLC45A1 | DLGAP2 | CACNA1I | NACAD | CAMK2N1 | PCLO | CABLES1 | AMZ1 | FKBP1B |
| NDRG3 | CEP170 | LRRC63 | RHEBL1 | ARRDC4 | TCEAL2 | PDXK | RASGRP1 | CYP46A1 | TNIK |
| PVALB | TTLL7 | MAST1 | PFN2 | TMEM200C | SERP2 | DPYSL2 | PCDH10 | PAAF1 | NECAB2 |
| PNMA3 | MAGEL2 | WFIKKN1 | PCP2 | RTL5 | KHDC1L | FOXH1 | SOX1 | ASPHD2 | SOX11 |
| GOLGA7B | HOXD1 | DNM3 | NRBP2 | LINGO1 | TMEM272 | MAP4 | LRFN1 | GRP | GRIA1 |
| KCNAB3 | NOXO1 | KLK6 | WSCD2 | PHACTR1 | SEMA6A | ILDR2 | PTPRN2 | STK32C | C5orf49 |
| TMEM169 | KBTBD11 | VGF | DLX2 | HTR1A | RNF182 | ZCCHC24 | TSR2 | CA8 | GLRA2 |
| NOVA1 | PPP3CA | RXFP1 | PDZD7 | CLDND1 | GAB2 | SYT9 | KCTD2 | TMEM59L | JPH4 |
| MICU3 | KCNIP4 | PPM1E | ANLN | NKX2-2 | NLGN2 | UNC13A | TCTE1 | NPB | FGFBP3 |
| ENPP6 | HS3ST2 | NEGR1 | NDST3 | DSCAM | RTKN | RASGEF1A | ST6GALNAC5 | KCNA4 | GABRA5 |
| RAB37 | KCTD8 | GABRA4 | MTMR10 | SYT4 | PPFIA3 | LRRTM2 | SLC1A2 | ATP9A | DTNBP1 |
| FAM131B | PPP1R3F | FAM234B | DBNDD1 | CHADL | GPR52 | KIF3A | SYN2 | CRYM | TMEM130 |
| RSPO2 | PRKCA | TBC1D12 | PLK5 | CHL1 | AMER2 | L3MBTL1 | GPR6 | SHISA9 | C20orf27 |
| RET | RNF220 | NSF | SLC10A4 | B3GALT2 | PCDHGA3 | LRRC10B | HPCA | TREM2 | DLL3 |
| CHN2 | AATK | NEUROD2 | KCNH3 | HSPA2 | SYNGR1 | PLCH2 | SLC25A22 | SOX2 | SCN9A |
| LGI3 | INSM2 | NAP1L3 | TNFRSF21 | ATP6V1G2 | NACC2 | VSX1 | HEPN1 | CTXN1 | GRID2 |
| ATRNL1 | MATK | IGSF21 | LARP6 | RALYL | ZNF488 | CRB2 | BTBD2 | SHANK2 | ELFN2 |
| STXBP5L | KALRN | CADPS | CNTNAP2 | PPP3R1 | ANKRD34A | NPTXR | ATP6V0E2 | SVOP | CGREF1 |
| SHD | C3orf80 | MAG | RTN4RL2 | SPOCK1 | ULBP1 | EN2 | B3GAT2 | TUNAR | GMFB |
| HR | KCNK12 | SHISA8 | BDNF | FBLL1 | MDGA2 | OPALIN | AGPAT5 | DPF1 | RHBDD2 |
| GRIN2A | JPT2 | MCF2L2 | SLC6A3 | PPP2R2B | DNAJC5 | VAX2 | TUBA1A | SLC1A3 | B3GAT1 |
| SYT12 | KCNJ6 | MAPK8IP3 | OPCML | APC | OR2H2 | MYO16 | TCEAL5 | FGFR3 | CPE |
| ABCG4 | CPEB1 | WNT7A | CAMK2A | OPRL1 | NFASC | KIRREL3 | ERBB4 | STUM | NUDT11 |
| GPR158 | MCHR1 | NRIP2 | PPP1R16B | LRFN2 | CDK5R2 | FAM171A2 | CACNB3 | CPNE7 | PLPPR2 |
| PRRT3 | ABCB9 | TRIM7 | CNTN2 | SPRED3 | HMX3 | CCNDBP1 | KLC1 | IL17D | KCNN3 |
| RFPL2 | SYNE1 | CBLN2 | FXYD7 | RAB33A | FAM222A | MEGF10 | TRIL | LRRC73 | THRA |
| SLC6A7 | SLC5A11 | BRINP2 | ISLR2 | RASGRP3 | RGS7BP | PRLH | ZIC4 | SEZ6 | PYDC1 |
| KCNT1 | DPP6 | MGAT4C | LHFPL4 | GPR19 | KIF1C | SOCS7 | HRK | GNG2 | HES5 |
| SATB2 | SNAP91 | IGDCC3 | CAMK2B | CAMK1G | RCOR2 | HIPK2 | DMTN | SHISAL1 | PALM |
| DCLK1 | RBFOX3 | SLC7A14 | SLC35F1 | TP73 | FOXB1 | GPR75 | SCN2A | DENND6B | LY6H |
| TBR1 | ANO8 | MADD | FAT3 | PRR7 | TUBG2 | GSX1 | GABRB3 | LRRN1 | NISCH |
| KLC2 | COL11A2 | HTR5A | BACE1 | AGBL4 | TMEM88B | NPY | PSAT1 | SLC25A18 | CELF4 |
| NPIPA8 | RASGRF1 | GNG13 | ESYT3 | WDR47 | FREM3 | CDK5R1 | PSD3 | TMEM255A | PNMT |
| CCDC177 | PLD5 | RTN3 | ELMOD1 | TMEM151B | KCNJ10 | LHPP | TMEM63C | CPLX3 | PDE2A |
| STXBP1 | SLC35F3 | KCNJ9 | CCDC136 | ATCAY | ANKRD13B | GLCE | KLHL35 | RAP1GAP | CCDC85C |
| PCSK1 | AMPH | CHRM1 | FAM163B | NGB | IGSF11 | GLRA3 | PCDH20 | SLC16A8 | DEAF1 |
| PRKCB | GPR162 | TPPP | APC2 | CNIH3 | KCNAB1 | KHDRBS2 | METRN | KCTD4 | GUK1 |
| DNASE1L2 | PTPRD | TIAM1 | NMNAT2 | KRTAP5-2 | KLHL2 | PKIB | PIN1 | ADCYAP1R1 | TMEM158 |
| QRFPR | MPPED1 | CLIP2 | PRRT2 | TTYH2 | CRLF1 | LY6G5C | DISP2 | CDH8 | CHCHD6 |
| RIMKLA | IGLON5 | FGFR2 | KLHL32 | EPHB1 | PCDHGC4 | CALM3 | ARPP19 | ASTN1 | PAK3 |
| ZNF385D | KANK4 | KCNN1 | LCNL1 | COL9A2 | CAMK4 | SLC2A13 | ARVCF | NCAM1 | PPP4R4 |
| GABRG2 | TRPC5 | CCDC88B | ZNF534 | CNP | POU3F4 | PTH2 | CDKL5 | RIMBP2 | SMARCD3 |
| FAM81A | TVP23A | NRXN1 | C1QL2 | CRYBB2 | CARTPT | KCNJ12 | OTOF | PTCHD1 | DIRAS3 |
| ATP2B2 | FNDC10 | GPR137C | TPRN | PCDHGC5 | ATP6V0A1 | CBX6 | SPRN | RUNX1T1 | FAM181A |
| LRFN5 | PLEKHG5 | BCL11A | TMEM151A | RGS11 | APBB3 | MMP17 | SCG3 | GPR88 | LYPD1 |
| CPLX1 | FMN2 | CLSTN2 | CHGB | GAS7 | HDAC11 | PURG | SAMD14 | GABRG1 | JPH3 |
| NEFH | CAMK1D | MBP | NCDN | MAPT | RAC3 | AP3B2 | NAT16 | RYR3 | MMD |
| KCNJ3 | CNNM1 | CLDN9 | C1QL3 | ASCL1 | NKAIN1 | DACH2 | PLEKHB1 | SLC25A48 | NKAIN3 |
| FAM216A | LZTS3 | SLC48A1 | COL9A3 | ALDOC | MAP6 | CBLN1 | SMOX | RUNDC3A | CENPVL3 |
| SLC24A2 | ADAM11 | SLC30A3 | NINJ2 | SIRT2 | KCNF1 | SIRPA | GABRA3 | ERC2 | FAM171B |
| RELL2 | CUX2 | NCALD | UGT8 | PRR36 | REM2 | GPR149 | COX6A1 | FLRT1 | ANKRD6 |
| CNTNAP1 | LIMK1 | ABTB2 | YWHAG | SKI | MAPK4 | TAC1 | PCDH19 | AGAP3 | GNG4 |
| SCAMP5 | CEND1 | TF | SLC39A10 | BAHCC1 | ABHD12B | PTGDS | ACBD7 | NRXN2 | CNIH2 |
| TMCC2 | NGEF | CDK18 | TRIM11 | KIAA1549L | SLC6A12 | AKAP5 | SLCO1C1 | ENHO | C11orf97 |
| ZNF365 | COL13A1 | LRRTM3 | GAL3ST1 | TMIE | SLC14A1 | SLC8A3 | SMIM29 | RAB3B | PLPPR4 |
| CDH7 | NPAS1 | UNC5D | KCNG3 | AP2A2 | NXPH4 | ABAT | TMPRSS5 | BEX1 | PLPPR3 |
| GLRB | KSR2 | CKMT1A | SYT1 | CACNA1G | MLLT11 | GDAP1L1 | GJB6 | VAT1L | LRRC3B |
| PIP5K1C | PPIP5K1 | ST18 | MYRF | SCN2B | ARHGAP23 | LRP1B | ASIC2 | BAIAP3 | MRAS |
| LRRC4 | KAZN | NEUROD6 | SYT13 | SYCE1 | PAK5 | FUT7 | SLITRK2 | ENKUR | C2orf80 |
| RHOBTB2 | GABRD | SEMA4D | DLX1 | TRIM41 | CABP7 | TMEM74B | SLC29A4 | SMIM10L2A | HPCAL4 |
| MCF2L | MAPKBP1 | SLC25A41 | CREG2 | MRAP2 | ASPHD1 | L1CAM | HMGCLL1 | TUBB2B | EFNB3 |
| CLEC2L | KCNV1 | CARNS1 | HS6ST2 | PCDH11Y | NPBWR1 | ATP8A2 | KCNQ3 | SOX3 | NKAIN4 |
| REEP2 | RBFOX1 | GPIHBP1 | GPR26 | NEUROG3 | PRR35 | HPCAL1 | DNAJB2 | SLC25A23 | GABRB1 |
| POU6F1 | MAP3K12 | PCDH9 | TMEM144 | CNTN5 | NTNG2 | CNTN1 | LILRA4 | CA11 | RIIAD1 |
| PEX5L | TIAM2 | HCN2 | CTXN3 | CKB | NRCAM | SYT6 | RAB40B | APLN |  |
| HECW1 | FSD1 | RIMS3 | NETO1 | SLC9A6 | OXT | DNAH9 | ENOPH1 | LCTL |  |
| CDR2L | HS3ST5 | SORCS1 | NALCN | ZDHHC22 | FAIM2 | KLHL1 | SLC7A10 | FBXL16 |  |
| NAP1L2 | SLC6A17 | LRRTM4 | MYT1L | KIF3C | KIF1A | SHISAL2A | SHC3 | PLPP4 |  |
| NT5M | COL27A1 | CDH18 | KCNIP2 | SCRT2 | PCDH11X | DPYSL4 | FUT9 | DTX1 |  |
| MICAL2 | TRIM9 | PARD6A | SNN | PTPRO | FFAR1 | C12orf76 | C2CD4C | STON2 |  |
| CPNE9 | TAS2R4 | PCDHA11 | NTM | RAB39A | RAB6B | ZIC1 | SORCS2 | SFXN5 |  |
| ANKS6 | GABRA1 | PTPN5 | ANK2 | NIM1K | ATOH7 | NDP | CDK5 | ZCCHC12 |  |
| FADS6 | PABPC1L2A | PTPRT | SHC4 | ACHE | TMEM160 | GNAO1 | VAMP2 | STOX1 |  |
| CLVS2 | KNDC1 | SLITRK1 | TMEM132B | CACNA1E | YWHAH | SNCG | ZIC5 | NXPH1 |  |
| SYT2 | MYO5A | ARPP21 | KIAA0513 | TMEM179 | SNX22 | CTXND1 | NAV3 | LRRTM1 |  |
| TESPA1 | PLCB4 | RPH3A | GDI1 | CCDC85B | CDH20 | SNX32 | NYAP2 | RAPGEF4 |  |
| ARC | KCNH4 | EHD3 | SCNN1D | LRRC4C | QKI | MARCKSL1 | PTPRN | GRIK3 |  |
| CELSR2 | RND1 | FGF14 | NKX6-2 | MTMR7 | MEIS3 | ADCY5 | FBXO44 | BEX2 |  |
| NXPH2 | BRINP1 | CHD5 | KISS1R | TRIM46 | ACOT7 | HEY1 | HABP4 | ECEL1 |  |
| NEFM | DAGLA | FAM124A | EGR4 | KCNH8 | HCN3 | WSCD1 | PCSK1N | GPR17 |  |
| IDS | MAP1LC3B2 | NPPC | GPR150 | GRIK4 | GRM2 | ZFR2 | RGS8 | KCNIP1 |  |
| LANCL1 | SRARP | CPLX2 | ZNF536 | CHRM5 | CDK2AP1 | FAM133A | YJEFN3 | GRIN2C |  |
| CADM2 | KCNH7 | KCNH5 | CERCAM | PAK1 | TRIM67 | SHF | RESP18 | CAMKV |  |
| ZNF519 | ZBTB18 | NPM2 | PNMA5 | GRIA2 | BEX5 | CCM2 | C10orf105 | TFPT |  |
| TMEM178A | PPFIA4 | PNMA6A | PIP4K2A | NOS1AP | FGF3 | STMN1 | RGS17 | CSPG5 |  |
| PAQR4 | FAM178B | SPP1 | HHATL | PRIMA1 | DCLK2 | GRM5 | GIT1 | PLPPR5 |  |
| SNTG1 | SMIM13 | ZNF653 | CHAT | C20orf204 | CPNE4 | NRXN3 | LHX2 | SCN3A |  |
| TRIM2 | GALNT9 | CSRNP3 | SBK1 | ARSF | OLFM3 | EVI5L | UCHL1 | ZNF521 |  |
| PCNX2 | KIF5A | PAQR3 | COLGALT2 | MOBP | THY1 | PCBP3 | TTC7B | ADGRL3 |  |
| EPHX4 | NIPA1 | MAPRE2 | CNTNAP4 | KIFC2 | ANKS1B | DLEU7 | ITM2C | CTNND2 |  |
| FLT3 | SFI1 | EML5 | CCKBR | FAM131A | AIF1L | MAGEE2 | GALNT17 | CXXC4 |  |
| VWC2 | B3GALT1 | TRH | SPIRE2 | SPOCK2 | SNORC | KIF1B | SCRG1 | RASL10A |  |
| KCNQ5 | NAPB | CIT | DOCK4 | CLIP3 | CHST2 | SALL2 | LRP3 | SOX21 |  |
| SLITRK3 | SV2A | DOCK10 | TTBK1 | FAM90A1 | BOK | ARL8A | SH3GL3 | TMEFF2 |  |
| SLC2A6 | KCNJ4 | MAP1A | STMN4 | C19orf73 | C1QTNF4 | GRIK1 | STRIP2 | SYNPR |  |
| ALS2 | MAP1B | TMEM121B | SEMA3B | CRTC1 | ATL1 | ASIC1 | PIGZ | NOVA2 |  |
| CCNI2 | RNF165 | SLC44A1 | ADGRL1 | MMP16 | SALL1 | CASKIN1 | TUBB2A | SNCA |  |
| KCNH1 | KCTD16 | ADORA1 | MVB12B | MGAT3 | CA10 | CCDC106 | MRO | ABR |  |
| SCN8A | ANKRD34C | DOCK3 | TLX3 | LHX6 | SYT7 | INSM1 | SLC8A2 | PNMA8A |  |
| STX1B | TMC7 | FAM219A | ESS2 | CHRNB2 | CNTFR | FAM241B | CDH10 | GPM6A |  |
| UNC13C | SOGA3 | SLC17A7 | MAP3K10 | ASPA | KCNE5 | PI4KA | TNR | SEMA6B |  |
| SYNJ2 | GABRB2 | RPRML | CLDN11 | SH3GL2 | BEST1 | CALY | PGM2L1 | DRD2 |  |
| SULT4A1 | PHYHIPL | ADCYAP1 | ASPDH | RASSF2 | RGS4 | PCYT1B | SPHKAP | STOML1 |  |
| ELAVL4 | SRGAP3 | CLVS1 | C9orf24 | SLC6A15 | LSAMP | DACT3 | NCAM2 | NKAIN2 |  |
| PGP | C11orf87 | PNLDC1 | CHST8 | PNMA8B | ADGRB1 | RNF175 | PIRT | CD200 |  |
| FBXW7 | GJC2 | SLX4 | PODXL2 | TYRO3 | LRTM2 | CADM3 | GFAP | MT3 |  |
| TRMT9B | ADD2 | PRR5L | FNDC9 | PRR18 | ZIC3 | FRMPD3 | TBC1D26 | LHFPL3 |  |
| CLCN4 | ZMAT4 | CACNB4 | CRH | NELL1 | CDIP1 | B4GALNT4 | MYRIP | CERS1 |  |
| SYT3 | STH | PRKACB | ITPKA | AK5 | ASRGL1 | HDC | ACSBG1 | PHACTR3 |  |
| APBA2 | RGS7 | B3GNT4 | FOXG1 | ADARB2 | CBFA2T3 | MEGF11 | TTC9B | SLC7A11 |  |
| SRGAP2 | ZEB2 | LRRC55 | MPP3 | PRRT1 | CHD7 | ZSCAN1 | C14orf132 | FSTL5 |  |
| AKAIN1 | CARMIL2 | NXPH3 | KCND2 | APLP1 | DCX | LGI1 | BRINP3 | FABP7 |  |
| KLHL4 | TRPC3 | CXXC5 | CDKN1C | PHYHIP | ARHGEF33 | GDA | APBA1 | GABBR1 |  |
| BRSK2 | SYBU | SRRM4 | DACT2 | PNMA2 | TCFL5 | KCNB2 | USP11 | CPNE5 |  |
| CAMKK2 | PTK2B | BTBD8 | NRSN2 | GAD1 | TSPAN7 | SSTR1 | CTXN2 | DOC2B |  |
| CACNG2 | SRRM3 | ABCC12 | IGIP | MAGI2 | POLB | GCSH | SIX3 | HSD11B1L | |
| CORO2B | DNAJC6 | OMG | GRM7 | GCK | AVP | HES7 | ARF3 | NPAS3 |  |
| CCDC184 | RNF144A | EXTL1 | DPF3 | RIMS4 | TLL1 | APBB1 | CORT | PIANP |  |
| TRAK2 | HS6ST3 | RIPPLY2 | GABBR2 | TCERG1L | KHDC1 | HMX1 | TMEM91 | COL9A1 |  |
| KCNIP3 | JAKMIP2 | GRIN1 | PDE1C | SLC6A1 | SAMD10 | CST3 | DZIP1 | LIX1 |  |
| FRMD5 | GRID1 | TMEM196 | PAQR8 | CRYGD | MAP2 | HECTD4 | PCDHGA12 | GLUD1 |  |
| CAMTA2 | SLC25A34 | GLDN | DAAM2 | EPHA5 | LRRC4B | PLD4 | SH2D5 | PDXP |  |
| BTBD3 | SV2B | KCNK9 | TCEAL7 | ACAP3 | ST6GAL2 | SLC32A1 | RELN | HHIPL1 |  |
| LYNX1 | GJB1 | DYNC1LI2 | NWD2 | UNC80 | TH | DPP10 | GPSM1 | ARHGAP36 | |
| RAP2A | VSTM2A | DGKZ | GRIA3 | ATP13A2 | BASP1 | ANKRD18B | NELL2 | NEXMIF |  |
| KCNAB2 | SEMA5B | IL11 | PCDHA5 | CNPY1 | OLIG2 | SLC1A6 | KIAA0319 | LUZP2 |  |
| ADCY1 | GALNT13 | GPR61 | LAMP5 | TMEM240 | SPOCK3 | PHF24 | RPS6KA4 | PACSIN1 |  |
| FAM135B | OLFM1 | SLITRK4 | SNPH | PADI2 | ARHGAP22 | DLGAP3 | NEURL1 | RSPO4 |  |
| SYNJ1 | HSPA12A | HOMER1 | DCC | RTN1 | SYP | DGKB | DPYSL5 | ARFGEF3 |  |
| UNC5A | KCND3 | SERPINI1 | OSTN | CDH9 | PDZD4 | HRH3 | EPOP | KLRC3 |  |
| ZFYVE28 | CHN1 | MORN1 | ERMN | TREML1 | CSAG1 | OLFM2 | GAREM2 | MLC1 |  |
| INA | ELAVL3 | ZNF821 | SYT16 | NECAB1 | KIAA0930 | RFTN2 | LMO3 | PCDH17 |  |
| TMEM266 | PACS2 | GPR37 | FAM13C | RADIL | WNT6 | SGSM1 | GAP43 | CHAD |  |
| CACNG7 | HAGHL | FCHO1 | EVI2A | ABHD8 | SLC22A17 | MAPK8IP2 | DRAXIN | PON2 |  |
| DSCAML1 | RPS6KL1 | NPY2R | ABCA3 | MOAP1 | KIF26B | CALN1 | TTYH3 | EPHA10 |  |
| KRT222 | SYN3 | ELOVL1 | LHX1 | HID1 | ICA1L | CHRM4 | ALK | ZCCHC17 |  |
| HTR2A | COL26A1 | KCNC2 | SEZ6L2 | RTN4RL1 | MTURN | PSD | CABCOCO1 | BEGAIN |  |
| HES6 | CALHM1 | OR14I1 | GAL3ST3 | CHRNA4 | DNER | VSTM2L | CTNNA2 | PANX2 |  |
| TSPYL4 | GFOD2 | MAMDC4 | DOC2A | C16orf86 | C2orf72 | NLGN1 | ARHGDIG | DDN |  |
| DRP2 | ATP1A3 | LZTS1 | NDRG4 | SRGAP2B | SSTR2 | SAMD4A | NAT14 | EMID1 |  |
| SCN4B | CLASP2 | KCNK10 | QDPR | SNX10 | TCEAL6 | SCD5 | CARMIL3 | KCNMB4 |  |
| SNCB | IPCEF1 | HHIP | TMEM74 | VN1R1 | ASIC3 | TMEM121 | GRIK5 | PTPRR |  |
| AMH | TRANK1 | NRGN | GRIA4 | SLC4A10 | GPRC5B | RASGEF1C | GPRIN1 | PPP1R9B |  |
| TMEM145 | HTR1E | DRD1 | TP53INP2 | BCAS1 | EXPH5 | NCS1 | GNG8 | SLC1A4 |  |

**Table S6.** The hub genes, their maximal clique centrality (MCC) values, and their annotations for each subtype.

| **Hub gene** | **MCC value** | **Annotation**a |
| --- | --- | --- |
| **Subtype 1** | | |
| **PLS1+** | | |
| SCN1A | 8 | Sodium channel protein type 1 subunit alpha; Mediates the voltage-dependent sodium ion permeability of excitable membranes. Plays a key role in the brain, **probably by regulating the moment when neurotransmitters are released in neurons.** |
| KCNAB3 | 8 | Voltage-gated potassium channel subunit beta-3; Accessory potassium channel protein which modulates the activity of the pore-forming alpha subunit. Alters the functional properties of Kv1.5. |
| KCNA1 | 8 | Potassium voltage-gated channel subfamily A member 1; Voltage-gated potassium channel that mediates transmembrane potassium transport. **Contributes to the regulation of the membrane potential and nerve signaling,** and prevents neuronal hyperexcitability. |
| **PLS1-** | | |
| GABBR1 | 13 | Gamma-aminobutyric acid type B receptor subunit 1; Component of a heterodimeric **G-protein coupled receptor for GABA**, formed by GABBR1 and GABBR2. |
| GABRB1 | 13 | Gamma-aminobutyric acid receptor subunit beta-1; Component of the heteropentameric **receptor for GABA**. |
| GABRG1 | 12 | Gamma-aminobutyric acid receptor subunit gamma-1; Mediates neuronal inhibition by binding to the **GABA/benzodiazepine receptor** and opening an integral chloride channel. |
| **Subtype 2** | | |
| **PLS1+** | | |
| SCN1A | 9 | See above. |
| SCN1B | 8 | Sodium channel subunit beta-1; Regulatory subunit of multiple voltage-gated sodium channel complexes that play important roles in excitable membranes in the brain, heart, and skeletal muscle. |
| KCNA1 | 8 | See above. |
| **PLS1-** | | |
| DLG4 | 26 | Disks large homolog 4; Interact with the cytoplasmic tail of NMDA receptor subunits and shaker-type potassium channels. Required for synaptic plasticity associated with NMDA receptor signaling. Overexpression or depletion of DLG4 **changes the ratio of excitatory to inhibitory synapses** in hippocampal neurons. May reduce the amplitude of ASIC3 acid-evoked currents by retaining the channel intracellularly. May regulate the intracellular trafficking of ADR1B. |
| GRIA1 | 24 | **Glutamate receptor** 1; Ionotropic glutamate receptor. L-glutamate acts as an excitatory neurotransmitter at many synapses in the central nervous system. |
| GRIN2B | 22 | **Glutamate receptor** ionotropic, NMDA 2B; Component of NMDA receptor complexes that function as heterotetrameric, ligand-gated ion channels with high calcium permeability and voltage-dependent sensitivity to magnesium. |

^a^ The annotations were extracted from Sting (<https://string-db.org/>).

**Table S7.** Associations between spatial patterns of aberrant functional connectivity in each subtype of major depressive disorder and neurotransmitter receptor/transporter densities.

| Names of R/T | Subtype 1 | | |  | Subtype 2 | | | Categories | References |
| --- | --- | --- | --- | --- | --- | --- | --- | --- | --- |
|  | *r* | *P* | *P_FDR_* |  | *r* | *P* | *P_FDR_* |  |  |
| 5-HT1a | 0.156 | 0.371 | 0.463 |  | 0.002 | 0.992 | 0.992 | Serotonin receptor | Savli 2012(Savli et al., 2012) |
| 5-HT1b | 0.218 | 0.102 | 0.167 |  | 0.356 | 0.025 | 0.064 | Serotonin receptor | Gallezot 2010(Folbergrová, Memezawa, Smith, & Siesjö, 1992), Savli 2012(Savli et al., 2012) |
| 5-HT2a | 0.320 | 0.001 | **0.009** |  | 0.288 | 0.001 | **0.018** | Serotonin receptor | Savli 2012(Savli et al., 2012) |
| 5-HT6 | 0.088 | 0.559 | 0.592 |  | 0.205 | 0.063 | 0.113 | Serotonin receptor | Radnakrishnan 2018(Radhakrishnan et al., 2018) |
| 5-HTT | -0.321 | 0.001 | **0.009** |  | -0.176 | 0.005 | **0.030** | Serotonin transporter | Fazio 2016(Fazio et al., 2016), Savli 2012(Savli et al., 2012) |
| CB1 | 0.354 | 0.018 | 0.065 |  | 0.388 | 0.018 | 0.063 | Cannabinoid receptor | Laurikainen 2018(Laurikainen et al., 2019), Normandin2015(Normandin et al., 2015) |
| D1 | -0.200 | 0.138 | 0.207 |  | -0.118 | 0.182 | 0.252 | Dopamine receptor | Kaller 2017(Kaller et al., 2017) |
| D2 | -0.226 | 0.055 | 0.162 |  | -0.144 | 0.039 | 0.086 | Dopamine receptor | Alarkurtti 2015(Alakurtti et al., 2015), Jaworska 2020(Jaworska et al., 2020), Sandiego 2015(Sandiego et al., 2015), Smith 2017(Smith et al., 2019) |
| DAT | -0.200 | 0.076 | 0.162 |  | -0.140 | 0.043 | 0.086 | Dopamine transporter | Dukart 2018(Dukart et al., 2018), Sasaki 2012(Sasaki et al., 2012) |
| GABAa | 0.258 | 0.002 | **0.012** |  | 0.148 | 0.021 | 0.063 | GABA receptor | Dukart 2018(Dukart et al., 2018) |
| H3 | -0.122 | 0.386 | 0.463 |  | 0.003 | 0.977 | 0.992 | Histamine receptor | Gallezot 2017(Gallezot et al., 2017) |
| M1 | 0.059 | 0.698 | 0.698 |  | 0.039 | 0.699 | 0.839 | Acetylcholine receptor | Naganawa 2020(Naganawa et al., 2021) |
| MOR | -0.323 | 0.097 | 0.167 |  | -0.037 | 0.901 | 0.992 | Mu-opioid receptor | Kantonen 2020(Kantonen et al., 2020), Turtonen2020(Turtonen et al., 2021) |
| NET | 0.110 | 0.454 | 0.511 |  | 0.427 | 0.002 | **0.018** | Norepinephrine transporter | Ding 2010(Ding et al., 2010), Hesse 2017(Hesse et al., 2017) |
| SV2A | 0.160 | 0.211 | 0.292 |  | 0.188 | 0.072 | 0.118 | Synapse marker | Finnema 2016(Finnema et al., 2018) |
| VAChT | -0.194 | 0.081 | 0.162 |  | -0.110 | 0.086 | 0.129 | Acetylcholine transporter | Aghourian 2017(Aghourian et al., 2017), bedard 2019(Bedard et al., 2019), Hansen 2021(Justine et al., 2021) |
| a4b2 | -0.328 | 0.007 | **0.032** |  | 0.144 | 0.238 | 0.306 | Acetylcholine receptor | Hillmer 2016(Hillmer et al., 2016) |
| mGluR5 | 0.243 | 0.070 | 0.162 |  | 0.306 | 0.009 | **0.041** | Glutamate receptor | Dubois2015(DuBois et al., 2016), Hansen 2021(Justine et al., 2021), Smart 2019(Smart et al., 2019) |

Abbreviation: R/T, receptor/transporter

**Table S8.** Associations between spatial patterns of aberrant functional connectivity patterns and meta-analytic cognitive functions.

| Index | Cognitive terms | Subtype 1 | | |  | Subtype 2 | | |
| --- | --- | --- | --- | --- | --- | --- | --- | --- |
|  |  | *r* | *P* | *P_FDR_* |  | *r* | *P* | *P_FDR_* |
| 1 | language | 0.16 | 0.281 | 0.601 |  | 0.33 | 0.074 | 0.178 |
| 2 | inference | 0.16 | 0.250 | 0.574 |  | 0.42 | 0.009 | 0.074 |
| 3 | interference | 0.05 | 0.715 | 0.905 |  | 0.32 | 0.077 | 0.178 |
| 4 | judgment | 0.08 | 0.563 | 0.815 |  | 0.26 | 0.144 | 0.238 |
| 5 | utility | -0.14 | 0.327 | 0.654 |  | 0.08 | 0.606 | 0.702 |
| 6 | task difficulty | 0.08 | 0.542 | 0.815 |  | 0.36 | 0.033 | 0.157 |
| 7 | speech perception | 0.33 | 0.001 | **0.031** |  | 0.43 | 0.004 | **0.048** |
| 8 | social cognition | -0.08 | 0.452 | 0.782 |  | 0.15 | 0.252 | 0.347 |
| 9 | hyperactivity | -0.09 | 0.474 | 0.805 |  | 0.15 | 0.386 | 0.493 |
| 10 | categorization | 0.35 | 0.001 | **0.031** |  | 0.48 | 0.002 | **0.048** |
| 11 | sleep | -0.12 | 0.250 | 0.574 |  | 0.10 | 0.373 | 0.482 |
| 12 | integration | 0.26 | 0.039 | 0.276 |  | 0.44 | 0.003 | **0.048** |
| 13 | recognition | 0.15 | 0.167 | 0.482 |  | 0.21 | 0.061 | 0.168 |
| 14 | adaptation | 0.20 | 0.050 | 0.295 |  | 0.32 | 0.005 | **0.048** |
| 15 | consciousness | 0.16 | 0.183 | 0.504 |  | 0.34 | 0.021 | 0.113 |
| 16 | skill | 0.27 | 0.070 | 0.321 |  | 0.45 | 0.013 | 0.095 |
| 17 | rule | -0.01 | 0.976 | 0.980 |  | 0.31 | 0.107 | 0.201 |
| 18 | object recognition | 0.31 | 0.035 | 0.271 |  | 0.11 | 0.641 | 0.729 |
| 19 | impulsivity | -0.29 | 0.047 | 0.291 |  | -0.04 | 0.826 | 0.875 |
| 20 | fixation | 0.24 | 0.152 | 0.463 |  | 0.26 | 0.220 | 0.317 |
| 21 | strength | 0.03 | 0.830 | 0.925 |  | 0.30 | 0.044 | 0.168 |
| 22 | stress | -0.20 | 0.109 | 0.397 |  | 0.00 | 0.994 | 1.000 |
| 23 | valence | -0.07 | 0.551 | 0.815 |  | 0.02 | 0.907 | 0.937 |
| 24 | emotion | -0.07 | 0.615 | 0.815 |  | 0.06 | 0.699 | 0.774 |
| 25 | sentence comprehension | 0.08 | 0.634 | 0.828 |  | 0.30 | 0.139 | 0.236 |
| 26 | perception | 0.28 | 0.008 | 0.165 |  | 0.39 | 0.003 | **0.048** |
| 27 | recall | -0.02 | 0.895 | 0.950 |  | 0.20 | 0.169 | 0.259 |
| 28 | intention | 0.05 | 0.609 | 0.815 |  | 0.28 | 0.058 | 0.168 |
| 29 | learning | -0.02 | 0.878 | 0.948 |  | 0.17 | 0.204 | 0.301 |
| 30 | manipulation | 0.13 | 0.338 | 0.665 |  | 0.33 | 0.049 | 0.168 |
| 31 | familiarity | 0.12 | 0.299 | 0.608 |  | 0.17 | 0.147 | 0.240 |
| 32 | addiction | -0.31 | 0.008 | 0.165 |  | -0.09 | 0.511 | 0.622 |
| 33 | arousal | -0.08 | 0.537 | 0.815 |  | 0.05 | 0.731 | 0.797 |
| 34 | induction | 0.08 | 0.517 | 0.815 |  | 0.26 | 0.060 | 0.168 |
| 35 | reinforcement learning | -0.20 | 0.070 | 0.321 |  | -0.06 | 0.507 | 0.622 |
| 36 | encoding | 0.06 | 0.565 | 0.815 |  | 0.15 | 0.152 | 0.243 |
| 37 | goal | 0.04 | 0.785 | 0.925 |  | 0.30 | 0.095 | 0.193 |
| 38 | motor control | 0.27 | 0.068 | 0.321 |  | 0.45 | 0.020 | 0.113 |
| 39 | visual attention | 0.26 | 0.095 | 0.380 |  | 0.32 | 0.126 | 0.217 |
| 40 | monitoring | 0.03 | 0.841 | 0.925 |  | 0.40 | 0.048 | 0.168 |
| 41 | reward anticipation | -0.27 | 0.017 | 0.186 |  | -0.18 | 0.027 | 0.140 |
| 42 | empathy | -0.10 | 0.454 | 0.782 |  | 0.18 | 0.365 | 0.476 |
| 43 | retention | 0.06 | 0.618 | 0.815 |  | 0.23 | 0.099 | 0.198 |
| 44 | spatial attention | 0.29 | 0.064 | 0.321 |  | 0.34 | 0.106 | 0.201 |
| 45 | knowledge | 0.09 | 0.501 | 0.815 |  | 0.33 | 0.029 | 0.144 |
| 46 | rhythm | 0.23 | 0.101 | 0.387 |  | 0.40 | 0.017 | 0.100 |
| 47 | psychosis | -0.05 | 0.695 | 0.898 |  | 0.28 | 0.056 | 0.168 |
| 48 | insight | -0.01 | 0.954 | 0.978 |  | 0.23 | 0.105 | 0.201 |
| 49 | localization | 0.39 | 0.001 | **0.031** |  | 0.50 | 0.001 | **0.048** |
| 50 | sustained attention | -0.04 | 0.781 | 0.925 |  | 0.23 | 0.170 | 0.259 |
| 51 | meaning | 0.16 | 0.293 | 0.606 |  | 0.31 | 0.079 | 0.178 |
| 52 | episodic memory | -0.03 | 0.782 | 0.925 |  | 0.08 | 0.454 | 0.574 |
| 53 | semantic memory | -0.08 | 0.600 | 0.815 |  | 0.09 | 0.659 | 0.736 |
| 54 | decision making | -0.18 | 0.209 | 0.540 |  | 0.10 | 0.593 | 0.694 |
| 55 | search | 0.07 | 0.556 | 0.815 |  | 0.23 | 0.086 | 0.184 |
| 56 | intelligence | -0.03 | 0.831 | 0.925 |  | 0.25 | 0.153 | 0.243 |
| 57 | listening | 0.24 | 0.061 | 0.321 |  | 0.41 | 0.012 | 0.093 |
| 58 | response inhibition | -0.11 | 0.433 | 0.778 |  | 0.26 | 0.186 | 0.278 |
| 59 | expertise | 0.25 | 0.017 | 0.186 |  | 0.29 | 0.005 | **0.048** |
| 60 | extinction | -0.11 | 0.344 | 0.667 |  | -0.07 | 0.576 | 0.687 |
| 61 | multisensory | 0.42 | 0.001 | **0.031** |  | 0.45 | 0.003 | **0.048** |
| 62 | maintenance | -0.02 | 0.879 | 0.948 |  | 0.28 | 0.123 | 0.215 |
| 63 | detection | 0.20 | 0.112 | 0.397 |  | 0.41 | 0.005 | **0.048** |
| 64 | association | 0.03 | 0.811 | 0.925 |  | 0.27 | 0.071 | 0.178 |
| 65 | discrimination | 0.18 | 0.124 | 0.416 |  | 0.38 | 0.004 | **0.048** |
| 66 | speech production | 0.30 | 0.012 | 0.186 |  | 0.42 | 0.005 | **0.048** |
| 67 | inhibition | -0.03 | 0.824 | 0.925 |  | 0.30 | 0.119 | 0.214 |
| 68 | word recognition | 0.23 | 0.069 | 0.321 |  | 0.29 | 0.065 | 0.175 |
| 69 | selective attention | 0.31 | 0.017 | 0.186 |  | 0.44 | 0.005 | **0.048** |
| 70 | belief | -0.04 | 0.773 | 0.925 |  | 0.24 | 0.171 | 0.259 |
| 71 | visual perception | 0.30 | 0.017 | 0.186 |  | 0.29 | 0.057 | 0.168 |
| 72 | thought | 0.02 | 0.904 | 0.950 |  | 0.31 | 0.053 | 0.168 |
| 73 | competition | 0.13 | 0.290 | 0.606 |  | 0.32 | 0.014 | 0.096 |
| 74 | pain | -0.05 | 0.750 | 0.925 |  | 0.25 | 0.208 | 0.303 |
| 75 | planning | 0.18 | 0.249 | 0.574 |  | 0.38 | 0.078 | 0.178 |
| 76 | action | 0.23 | 0.117 | 0.403 |  | 0.38 | 0.041 | 0.168 |
| 77 | context | 0.03 | 0.807 | 0.925 |  | 0.27 | 0.084 | 0.184 |
| 78 | loss | -0.11 | 0.369 | 0.693 |  | 0.08 | 0.589 | 0.694 |
| 79 | imagery | 0.30 | 0.043 | 0.281 |  | 0.41 | 0.038 | 0.168 |
| 80 | timing | 0.19 | 0.153 | 0.463 |  | 0.41 | 0.008 | 0.071 |
| 81 | rehearsal | 0.21 | 0.137 | 0.447 |  | 0.36 | 0.048 | 0.168 |
| 82 | balance | 0.00 | 0.980 | 0.980 |  | 0.30 | 0.123 | 0.215 |
| 83 | coordination | 0.25 | 0.093 | 0.380 |  | 0.39 | 0.052 | 0.168 |
| 84 | strategy | -0.05 | 0.710 | 0.905 |  | 0.28 | 0.106 | 0.201 |
| 85 | attention | 0.16 | 0.222 | 0.562 |  | 0.39 | 0.017 | 0.100 |
| 86 | decision | -0.09 | 0.515 | 0.815 |  | 0.17 | 0.359 | 0.474 |
| 87 | cognitive control | -0.07 | 0.615 | 0.815 |  | 0.30 | 0.116 | 0.212 |
| 88 | movement | 0.30 | 0.040 | 0.276 |  | 0.42 | 0.039 | 0.168 |
| 89 | expectancy | -0.12 | 0.359 | 0.685 |  | 0.18 | 0.252 | 0.347 |
| 90 | emotion regulation | -0.11 | 0.415 | 0.757 |  | 0.09 | 0.655 | 0.736 |
| 91 | concept | 0.12 | 0.451 | 0.782 |  | 0.34 | 0.074 | 0.178 |
| 92 | memory | 0.01 | 0.931 | 0.962 |  | 0.24 | 0.079 | 0.178 |
| 93 | autobiographical memory | -0.22 | 0.023 | 0.219 |  | -0.01 | 0.880 | 0.925 |
| 94 | uncertainty | -0.20 | 0.147 | 0.463 |  | 0.18 | 0.311 | 0.424 |
| 95 | anxiety | -0.15 | 0.192 | 0.518 |  | -0.03 | 0.820 | 0.875 |
| 96 | memory retrieval | -0.16 | 0.157 | 0.464 |  | 0.12 | 0.340 | 0.453 |
| 97 | response selection | 0.09 | 0.516 | 0.815 |  | 0.38 | 0.060 | 0.168 |
| 98 | navigation | 0.02 | 0.896 | 0.950 |  | 0.02 | 0.933 | 0.956 |
| 99 | consolidation | -0.02 | 0.843 | 0.925 |  | 0.00 | 1.000 | 1.000 |
| 100 | working memory | 0.07 | 0.585 | 0.815 |  | 0.38 | 0.046 | 0.168 |
| 101 | distraction | 0.03 | 0.798 | 0.925 |  | 0.18 | 0.237 | 0.334 |
| 102 | reading | 0.23 | 0.103 | 0.387 |  | 0.29 | 0.092 | 0.190 |
| 103 | facial expression | 0.13 | 0.233 | 0.574 |  | 0.06 | 0.512 | 0.622 |
| 104 | effort | 0.05 | 0.743 | 0.925 |  | 0.35 | 0.050 | 0.168 |
| 105 | reasoning | -0.03 | 0.834 | 0.925 |  | 0.31 | 0.086 | 0.184 |
| 106 | risk | -0.22 | 0.079 | 0.338 |  | 0.05 | 0.733 | 0.797 |
| 107 | retrieval | 0.00 | 0.978 | 0.980 |  | 0.20 | 0.167 | 0.259 |
| 108 | anticipation | -0.26 | 0.031 | 0.256 |  | -0.04 | 0.749 | 0.808 |
| 109 | language comprehension | 0.13 | 0.414 | 0.757 |  | 0.30 | 0.142 | 0.238 |
| 110 | priming | 0.25 | 0.018 | 0.186 |  | 0.32 | 0.005 | **0.048** |
| 111 | face recognition | 0.14 | 0.265 | 0.587 |  | -0.09 | 0.564 | 0.679 |
| 112 | communication | 0.15 | 0.236 | 0.574 |  | 0.34 | 0.017 | 0.100 |
| 113 | mental imagery | 0.19 | 0.199 | 0.525 |  | 0.30 | 0.089 | 0.187 |
| 114 | morphology | 0.10 | 0.558 | 0.815 |  | 0.23 | 0.327 | 0.441 |
| 115 | verbal fluency | 0.08 | 0.593 | 0.815 |  | 0.30 | 0.114 | 0.211 |
| 116 | mood | -0.17 | 0.174 | 0.490 |  | -0.02 | 0.901 | 0.937 |
| 117 | naming | 0.30 | 0.029 | 0.256 |  | 0.34 | 0.060 | 0.168 |
| 118 | eating | -0.13 | 0.273 | 0.594 |  | 0.00 | 0.995 | 1.000 |
| 119 | updating | 0.01 | 0.926 | 0.962 |  | 0.31 | 0.071 | 0.178 |
| 120 | fear | -0.08 | 0.487 | 0.815 |  | -0.06 | 0.630 | 0.723 |
| 121 | focus | 0.07 | 0.591 | 0.815 |  | 0.32 | 0.045 | 0.168 |
| 122 | salience | -0.13 | 0.262 | 0.587 |  | 0.11 | 0.490 | 0.614 |
| 123 | efficiency | 0.07 | 0.605 | 0.815 |  | 0.31 | 0.076 | 0.178 |
| 124 | gaze | 0.24 | 0.073 | 0.323 |  | 0.20 | 0.231 | 0.329 |

**Table S9.** Shared and subtype-2-specific cognitive classifications, and corresponding terms&annotations belonging to them.

|  | **Classification** | **Term** | **Annotation^a^** |
| --- | --- | --- | --- |
| Shared by two subtypes | language | speech perception | The process by which the sounds of language are heard, interpreted and understood. |
|  |  | speech production | The process by which spoken words are selected to be produced, have their phonetics formulated and then finally are articulated by the motor system in the vocal apparatus. Speech production can be spontaneous such as when a person creates the words of a conversation, reaction such as when they name a picture or read aloud a written word, or a vocal imitation such as in speech repetition. Speech production is not the same as language production since language can also be produced manually by signs. |
|  | reasoning and decision making | categorization | The assignment of a stimulus to one of a set of categories. |
|  | perception | localization | Part of perception and consists of visual, auditory, and spatial localization. |
|  |  | discrimination | Responding differently to stimuli that differ in some aspect. |
|  |  | integration | Coordination of mental processes into a normal effective personality or with the individual's environment. |
|  |  | perception | The conscious experience or mental registration of a sensory stimulus. |
|  |  | detection | To determine the presence of a stimulus. |
|  | - | multisensory | A process associated with multiple sensory modalities. |
| Specific for subtype 2 | learning and memory | adaptation | Adjustment to environmental conditions; adjustment of a sense organ to the intensity or quality of stimulation; modification of an organism or its parts that makes it more fit for existence under the conditions of its environment. |
|  |  | expertise | Having a highly cultivated level of skill in a particular domain. Occurs after prolonged experience in a domain. |
|  |  | priming | Priming is the effect of prior exposure to a somehow (e.g. perceptually or semantically) related stimulus on the response to a subsequent stimulus. This effect may be positive and facilitatory (e.g. naming of an object is typically faster when that object has already been recently named) or negative and detrimental (e.g. slower response to a previously ignored stimulus). |
|  | attention | selective attention | When multiple external sensory inputs are present, the process of dedicating cognitive and perceptual resources to one type/set of input and attenuating receptiveness to other inputs. |

^a^ The annotations were extracted from the cognitive atlas (<https://cognitiveatlas.org/>)

**Figure S1.** Sample selection.

**
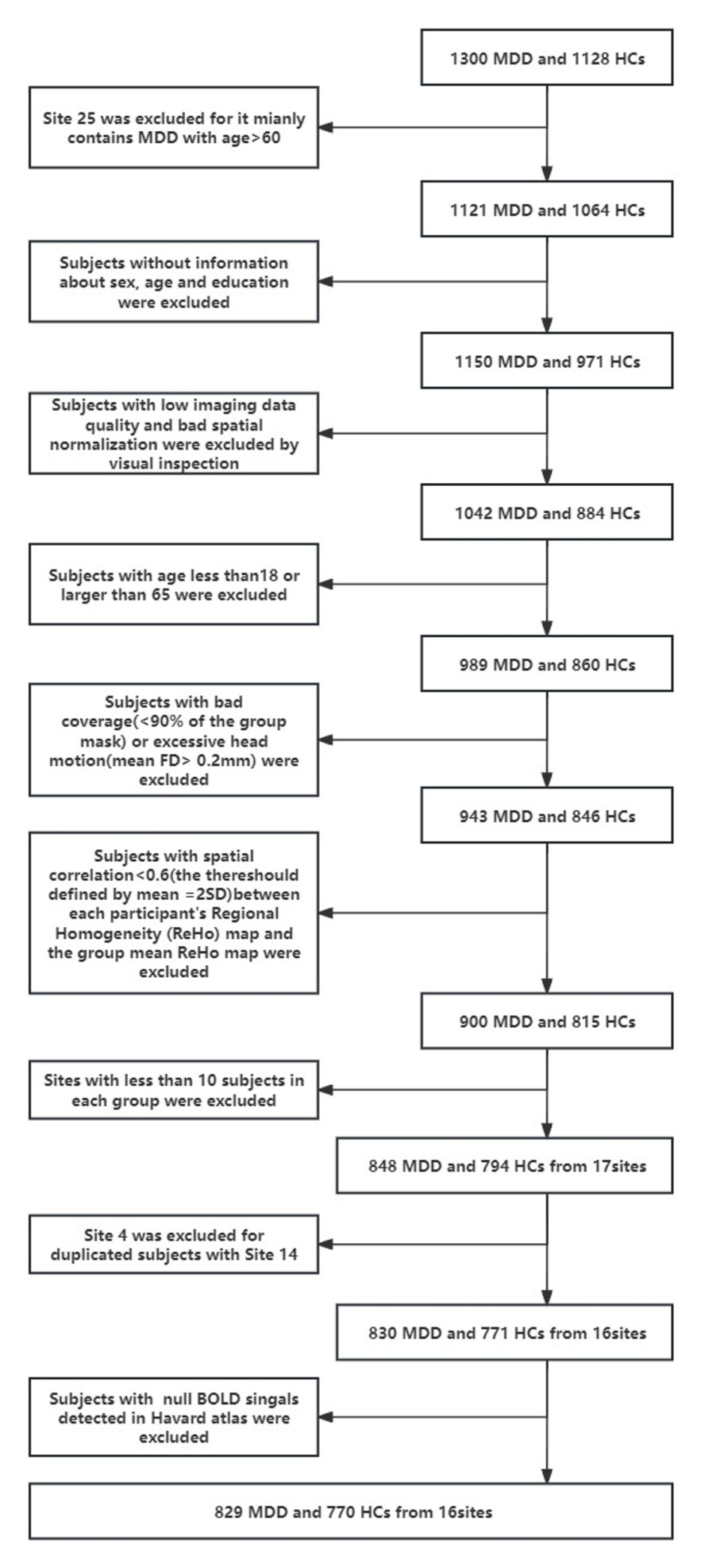
**

Abbreviation: MDD, major depressive disorder; HC, healthy controls; FD, framewise displacement; SD, standard deviation; AAL, Anatomical Automatic Labeling.

**Figure S2.** The two major MDD subtypes were identified by multi-resolution hierarchical clustering.


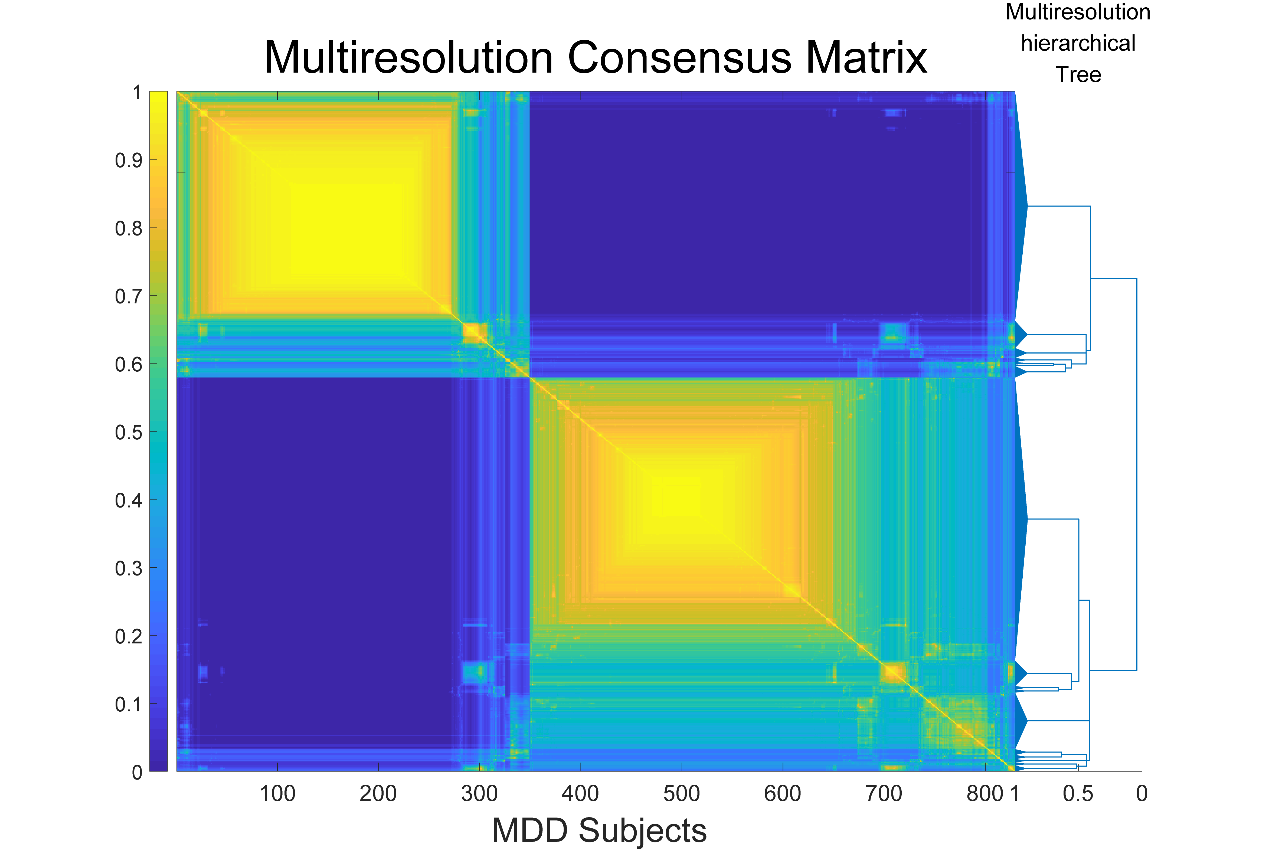


Abbreviation: MDD, major depressive disorder.

**Figure S3. Associations between functional connectivity (FC) alterations and cognitions for two subtypes of major depressive disorder.** Wordcloud plot showed cognitive associations with FC alternations with a correlation coefficient (*r*) less than 0.1. If *r* was less than 0.1, 0.05, or 0.05 with false-discovery rate correction, the corresponding cognition was marked as gray, light purple, and dark red, respectively.


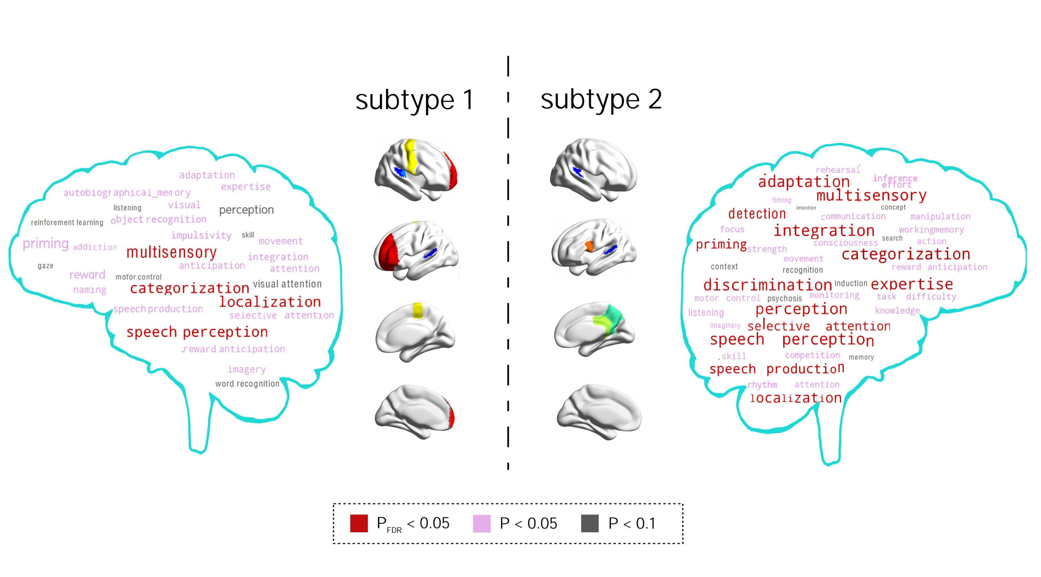


**References:**

Aghourian, M., Legault-Denis, C., Soucy, J. P., Rosa-Neto, P., Gauthier, S., Kostikov, A., . . . Bédard, M. A. (2017). Quantification of brain cholinergic denervation in Alzheimer's disease using PET imaging with [(18)F]-FEOBV. *Mol Psychiatry, 22*(11), 1531-1538. doi:10.1038/mp.2017.183

Alakurtti, K., Johansson, J. J., Joutsa, J., Laine, M., Bäckman, L., Nyberg, L., & Rinne, J. O. (2015). Long-term test-retest reliability of striatal and extrastriatal dopamine D2/3 receptor binding: study with [(11)C]raclopride and high-resolution PET. *J Cereb Blood Flow Metab, 35*(7), 1199-1205. doi:10.1038/jcbfm.2015.53

Arnatkeviciute, A., Fulcher, B. D., & Fornito, A. (2019). A practical guide to linking brain-wide gene expression and neuroimaging data. *Neuroimage, 189*, 353-367. doi:10.1016/j.neuroimage.2019.01.011

Bedard, M. A., Aghourian, M., Legault-Denis, C., Postuma, R. B., Soucy, J. P., Gagnon, J. F., . . . Montplaisir, J. (2019). Brain cholinergic alterations in idiopathic REM sleep behaviour disorder: a PET imaging study with (18)F-FEOBV. *Sleep Med, 58*, 35-41. doi:10.1016/j.sleep.2018.12.020

Chin, C. H., Chen, S. H., Wu, H. H., Ho, C. W., Ko, M. T., & Lin, C. Y. (2014). cytoHubba: identifying hub objects and sub-networks from complex interactome. *BMC Syst Biol, 8 Suppl 4*(Suppl 4), S11. doi:10.1186/1752-0509-8-s4-s11

Ding, Y. S., Singhal, T., Planeta-Wilson, B., Gallezot, J. D., Nabulsi, N., Labaree, D., . . . Malison, R. T. (2010). PET imaging of the effects of age and cocaine on the norepinephrine transporter in the human brain using (S,S)-[(11)C]O-methylreboxetine and HRRT. *Synapse, 64*(1), 30-38. doi:10.1002/syn.20696

DuBois, J. M., Rousset, O. G., Rowley, J., Porras-Betancourt, M., Reader, A. J., Labbe, A., . . . Kobayashi, E. (2016). Characterization of age/sex and the regional distribution of mGluR5 availability in the healthy human brain measured by high-resolution [(11)C]ABP688 PET. *Eur J Nucl Med Mol Imaging, 43*(1), 152-162. doi:10.1007/s00259-015-3167-6

Dukart, J., Holiga, Š., Chatham, C., Hawkins, P., Forsyth, A., McMillan, R., . . . Sambataro, F. (2018). Cerebral blood flow predicts differential neurotransmitter activity. *Sci Rep, 8*(1), 4074. doi:10.1038/s41598-018-22444-0

Fazio, P., Schain, M., Varnäs, K., Halldin, C., Farde, L., & Varrone, A. (2016). Mapping the distribution of serotonin transporter in the human brainstem with high-resolution PET: Validation using postmortem autoradiography data. *Neuroimage, 133*, 313-320. doi:10.1016/j.neuroimage.2016.03.019

Finnema, S. J., Nabulsi, N. B., Mercier, J., Lin, S. F., Chen, M. K., Matuskey, D., . . . Carson, R. E. (2018). Kinetic evaluation and test-retest reproducibility of [(11)C]UCB-J, a novel radioligand for positron emission tomography imaging of synaptic vesicle glycoprotein 2A in humans. *J Cereb Blood Flow Metab, 38*(11), 2041-2052. doi:10.1177/0271678x17724947

Folbergrová, J., Memezawa, H., Smith, M. L., & Siesjö, B. K. (1992). Focal and perifocal changes in tissue energy state during middle cerebral artery occlusion in normo- and hyperglycemic rats. *J Cereb Blood Flow Metab, 12*(1), 25-33. doi:10.1038/jcbfm.1992.4

Fulcher, B. D., Arnatkeviciute, A., & Fornito, A. (2021). Overcoming false-positive gene-category enrichment in the analysis of spatially resolved transcriptomic brain atlas data. *Nat Commun, 12*(1), 2669. doi:10.1038/s41467-021-22862-1

Gallezot, J. D., Planeta, B., Nabulsi, N., Palumbo, D., Li, X., Liu, J., . . . Carson, R. E. (2017). Determination of receptor occupancy in the presence of mass dose: [(11)C]GSK189254 PET imaging of histamine H(3) receptor occupancy by PF-03654746. *J Cereb Blood Flow Metab, 37*(3), 1095-1107. doi:10.1177/0271678x16650697

Gaudet, P., Logie, C., Lovering, R. C., Kuiper, M., Lægreid, A., & Thomas, P. D. (2021). Gene Ontology representation for transcription factor functions. *Biochim Biophys Acta Gene Regul Mech, 1864*(11-12), 194752. doi:10.1016/j.bbagrm.2021.194752

Hawrylycz, M. J., Lein, E. S., Guillozet-Bongaarts, A. L., Shen, E. H., Ng, L., Miller, J. A., . . . Jones, A. R. (2012). An anatomically comprehensive atlas of the adult human brain transcriptome. *Nature, 489*(7416), 391-399. doi:10.1038/nature11405

Hesse, S., Becker, G. A., Rullmann, M., Bresch, A., Luthardt, J., Hankir, M. K., . . . Sabri, O. (2017). Central noradrenaline transporter availability in highly obese, non-depressed individuals. *Eur J Nucl Med Mol Imaging, 44*(6), 1056-1064. doi:10.1007/s00259-016-3590-3

Hillmer, A. T., Esterlis, I., Gallezot, J. D., Bois, F., Zheng, M. Q., Nabulsi, N., . . . Cosgrove, K. P. (2016). Imaging of cerebral α4β2* nicotinic acetylcholine receptors with (-)-[(18)F]Flubatine PET: Implementation of bolus plus constant infusion and sensitivity to acetylcholine in human brain. *Neuroimage, 141*, 71-80. doi:10.1016/j.neuroimage.2016.07.026

Jaworska, N., Cox, S. M. L., Tippler, M., Castellanos-Ryan, N., Benkelfat, C., Parent, S., . . . Leyton, M. (2020). Extra-striatal D(2/3) receptor availability in youth at risk for addiction. *Neuropsychopharmacology, 45*(9), 1498-1505. doi:10.1038/s41386-020-0662-7

Jeub, L. G. S., Sporns, O., & Fortunato, S. (2018). Multiresolution Consensus Clustering in Networks. *Sci Rep, 8*(1), 3259. doi:10.1038/s41598-018-21352-7

Justine, Y. H., Golia, S., Ross, D. M., Kelly, S., Sylvia, M. L. C., Yanjun, W., . . . Bratislav, M. (2021). Mapping neurotransmitter systems to the structural and functional organization of the human neocortex. *bioRxiv*, 2021.2010.2028.466336. doi:10.1101/2021.10.28.466336

Kaller, S., Rullmann, M., Patt, M., Becker, G. A., Luthardt, J., Girbardt, J., . . . Sabri, O. (2017). Test-retest measurements of dopamine D(1)-type receptors using simultaneous PET/MRI imaging. *Eur J Nucl Med Mol Imaging, 44*(6), 1025-1032. doi:10.1007/s00259-017-3645-0

Kantonen, T., Karjalainen, T., Isojärvi, J., Nuutila, P., Tuisku, J., Rinne, J., . . . Nummenmaa, L. (2020). Interindividual variability and lateralization of μ-opioid receptors in the human brain. *Neuroimage, 217*, 116922. doi:10.1016/j.neuroimage.2020.116922

Kaufman, L., & Rousseeuw, P. J. (2009). *Finding groups in data: an introduction to cluster analysis*: John Wiley & Sons.

Laurikainen, H., Tuominen, L., Tikka, M., Merisaari, H., Armio, R. L., Sormunen, E., . . . Hietala, J. (2019). Sex difference in brain CB1 receptor availability in man. *Neuroimage, 184*, 834-842. doi:10.1016/j.neuroimage.2018.10.013

Makris, N., Meyer, J. W., Bates, J. F., Yeterian, E. H., Kennedy, D. N., & Caviness, V. S. (1999). MRI-Based topographic parcellation of human cerebral white matter and nuclei II. Rationale and applications with systematics of cerebral connectivity. *Neuroimage, 9*(1), 18-45. doi:10.1006/nimg.1998.0384

Martino, E., Chiarugi, S., Margheriti, F., & Garau, G. (2021). Mapping, Structure and Modulation of PPI. *Front Chem, 9*, 718405. doi:10.3389/fchem.2021.718405

Naganawa, M., Nabulsi, N., Henry, S., Matuskey, D., Lin, S. F., Slieker, L., . . . Huang, Y. (2021). First-in-Human Assessment of (11)C-LSN3172176, an M1 Muscarinic Acetylcholine Receptor PET Radiotracer. *J Nucl Med, 62*(4), 553-560. doi:10.2967/jnumed.120.246967

Newman, M. E., & Girvan, M. (2004). Finding and evaluating community structure in networks. *Phys Rev E Stat Nonlin Soft Matter Phys, 69*(2 Pt 2), 026113. doi:10.1103/PhysRevE.69.026113

Normandin, M. D., Zheng, M. Q., Lin, K. S., Mason, N. S., Lin, S. F., Ropchan, J., . . . Huang, Y. (2015). Imaging the cannabinoid CB1 receptor in humans with [11C]OMAR: assessment of kinetic analysis methods, test-retest reproducibility, and gender differences. *J Cereb Blood Flow Metab, 35*(8), 1313-1322. doi:10.1038/jcbfm.2015.46

Power, J. D., Cohen, A. L., Nelson, S. M., Wig, G. S., Barnes, K. A., Church, J. A., . . . Petersen, S. E. (2011). Functional network organization of the human brain. *Neuron, 72*(4), 665-678. doi:10.1016/j.neuron.2011.09.006

Radhakrishnan, R., Nabulsi, N., Gaiser, E., Gallezot, J. D., Henry, S., Planeta, B., . . . Matuskey, D. (2018). Age-Related Change in 5-HT(6) Receptor Availability in Healthy Male Volunteers Measured with (11)C-GSK215083 PET. *J Nucl Med, 59*(9), 1445-1450. doi:10.2967/jnumed.117.206516

Rasero, J., Diez, I., Cortes, J. M., Marinazzo, D., & Stramaglia, S. (2019). Connectome sorting by consensus clustering increases separability in group neuroimaging studies. *Netw Neurosci, 3*(2), 325-343. doi:10.1162/netn_a_00074

Rasero, J., Jimenez-Marin, A., Diez, I., Toro, R., Hasan, M. T., & Cortes, J. M. (2023). The Neurogenetics of Functional Connectivity Alterations in Autism: Insights From Subtyping in 657 Individuals. *Biol Psychiatry*. doi:10.1016/j.biopsych.2023.04.014

Rasero, J., Pellicoro, M., Angelini, L., Cortes, J. M., Marinazzo, D., & Stramaglia, S. (2017). Consensus clustering approach to group brain connectivity matrices. *Netw Neurosci, 1*(3), 242-253. doi:10.1162/NETN_a_00017

Reichardt, J., & Bornholdt, S. (2006). Statistical mechanics of community detection. *Phys Rev E Stat Nonlin Soft Matter Phys, 74*(1 Pt 2), 016110. doi:10.1103/PhysRevE.74.016110

Ross, D. M., Aurina, A., Jean-Baptiste, P., Ben, D. F., Alex, F., & Bratislav, M. (2021). Standardizing workflows in imaging transcriptomics with the abagen toolbox. *bioRxiv*, 2021.2007.2008.451635. doi:10.1101/2021.07.08.451635

Sandiego, C. M., Gallezot, J. D., Lim, K., Ropchan, J., Lin, S. F., Gao, H., . . . Cosgrove, K. P. (2015). Reference region modeling approaches for amphetamine challenge studies with [11C]FLB 457 and PET. *J Cereb Blood Flow Metab, 35*(4), 623-629. doi:10.1038/jcbfm.2014.237

Sasaki, T., Ito, H., Kimura, Y., Arakawa, R., Takano, H., Seki, C., . . . Suhara, T. (2012). Quantification of dopamine transporter in human brain using PET with 18F-FE-PE2I. *J Nucl Med, 53*(7), 1065-1073. doi:10.2967/jnumed.111.101626

Savli, M., Bauer, A., Mitterhauser, M., Ding, Y. S., Hahn, A., Kroll, T., . . . Lanzenberger, R. (2012). Normative database of the serotonergic system in healthy subjects using multi-tracer PET. *Neuroimage, 63*(1), 447-459. doi:10.1016/j.neuroimage.2012.07.001

Shafiei, G., Markello, R. D., Makowski, C., Talpalaru, A., Kirschner, M., Devenyi, G. A., . . . Misic, B. (2020). Spatial Patterning of Tissue Volume Loss in Schizophrenia Reflects Brain Network Architecture. *Biol Psychiatry, 87*(8), 727-735. doi:10.1016/j.biopsych.2019.09.031

Shehzad, Z., Kelly, C., Reiss, P. T., Cameron Craddock, R., Emerson, J. W., McMahon, K., . . . Milham, M. P. (2014). A multivariate distance-based analytic framework for connectome-wide association studies. *Neuroimage, 93 Pt 1*(0 1), 74-94. doi:10.1016/j.neuroimage.2014.02.024

Smart, K., Cox, S. M. L., Scala, S. G., Tippler, M., Jaworska, N., Boivin, M., . . . Leyton, M. (2019). Sex differences in [(11)C]ABP688 binding: a positron emission tomography study of mGlu5 receptors. *Eur J Nucl Med Mol Imaging, 46*(5), 1179-1183. doi:10.1007/s00259-018-4252-4

Smith, C. T., Crawford, J. L., Dang, L. C., Seaman, K. L., San Juan, M. D., Vijay, A., . . . Samanez-Larkin, G. R. (2019). Partial-volume correction increases estimated dopamine D2-like receptor binding potential and reduces adult age differences. *J Cereb Blood Flow Metab, 39*(5), 822-833. doi:10.1177/0271678x17737693

Turtonen, O., Saarinen, A., Nummenmaa, L., Tuominen, L., Tikka, M., Armio, R. L., . . . Hietala, J. (2021). Adult Attachment System Links With Brain Mu Opioid Receptor Availability In Vivo. *Biol Psychiatry Cogn Neurosci Neuroimaging, 6*(3), 360-369. doi:10.1016/j.bpsc.2020.10.013

Wei, W., Deng, L., Qiao, C., Yin, Y., Zhang, Y., Li, X., . . . Li, T. (2023). Neural variability in three major psychiatric disorders. *Mol Psychiatry*. doi:10.1038/s41380-023-02164-2

Whitaker, K. J., Vértes, P. E., Romero-Garcia, R., Váša, F., Moutoussis, M., Prabhu, G., . . . Bullmore, E. T. (2016). Adolescence is associated with genomically patterned consolidation of the hubs of the human brain connectome. *Proc Natl Acad Sci U S A, 113*(32), 9105-9110. doi:10.1073/pnas.1601745113

Yan, C. G., Chen, X., Li, L., Castellanos, F. X., Bai, T. J., Bo, Q. J., . . . Zang, Y. F. (2019). Reduced default mode network functional connectivity in patients with recurrent major depressive disorder. *Proc Natl Acad Sci U S A, 116*(18), 9078-9083. doi:10.1073/pnas.1900390116

Yarkoni, T., Poldrack, R. A., Nichols, T. E., Van Essen, D. C., & Wager, T. D. (2011). Large-scale automated synthesis of human functional neuroimaging data. *Nat Methods, 8*(8), 665-670. doi:10.1038/nmeth.1635

Zhou, Y., Zhou, B., Pache, L., Chang, M., Khodabakhshi, A. H., Tanaseichuk, O., . . . Chanda, S. K. (2019). Metascape provides a biologist-oriented resource for the analysis of systems-level datasets. *Nat Commun, 10*(1), 1523. doi:10.1038/s41467-019-09234-6
